# RID is required for both repeat-induced point mutation and nucleation of a novel transitional heterochromatic state for euchromatic repeats

**DOI:** 10.1093/nar/gkaf263

**Published:** 2025-04-04

**Authors:** Zhen He, Nannan Wu, Ruonan Yao, Huawei Tan, Yingying Sun, Jingxuan Chen, Lan Xue, Xiaonan Chen, Sihai Yang, Laurence D Hurst, Long Wang, Ju Huang

**Affiliations:** School of Life Sciences, Nanjing University, Nanjing 210023, China; School of Life Sciences, Nanjing University, Nanjing 210023, China; School of Life Sciences, Nanjing University, Nanjing 210023, China; School of Life Sciences, Nanjing University, Nanjing 210023, China; School of Life Sciences, Nanjing University, Nanjing 210023, China; School of Life Sciences, Nanjing University, Nanjing 210023, China; School of Life Sciences, Nanjing University, Nanjing 210023, China; School of Life Sciences, Nanjing University, Nanjing 210023, China; School of Life Sciences, Nanjing University, Nanjing 210023, China; Co-Innovation Center for Sustainable Forestry in Southern China, Nanjing Forestry University, Nanjing, Jiangsu 210000, China; State Key Laboratory of Pharmaceutical Biotechnology, Nanjing University, Nanjing 210023, China; Milner Centre for Evolution, Department of Life Sciences, University of Bath, Bath, BA2 7AY, UK; School of Life Sciences, Nanjing University, Nanjing 210023, China; State Key Laboratory of Pharmaceutical Biotechnology, Nanjing University, Nanjing 210023, China; State Key Laboratory of Crop Genetics and Germplasm Enhancement, Bioinformatics Center, Academy for Advanced Interdisciplinary Studies, Nanjing Agricultural University, Nanjing 210095, China

## Abstract

To maintain genome integrity, repeat sequences are subject to heterochromatin inactivation and, in *Neurospora*, repeat-induced point mutation (RIP). The initiating factors behind both are poorly understood. We resolve the paradoxical observation that newly introduced Repeat-Linker-Repeat (R-L-R) constructs require RID alone for RIP, while genomic repeats are RIPed in the absence of RID, showing that eu- and hetero- chromatic repeats are handled differently, the latter additionally requiring DIM-2. The differences between mechanisms associated with older and newer duplicates caution against extrapolation from mechanisms inferred from model experimental systems. Additionally, while chromatin status affects RIP, we also show that RID, when tethered with LexA, acts as a nucleation center for the transition from euchromatin to heterochromatin in an HDA-1 dependent fashion. Constitutive heterochromatin by contrast is largely HDA1 independent and depends on HDA-1 paralogs. RID is thus a dual function initiator of both RIP and the transition to heterochromatin.

## Graphical Abstract

**Graphical Abstract:**
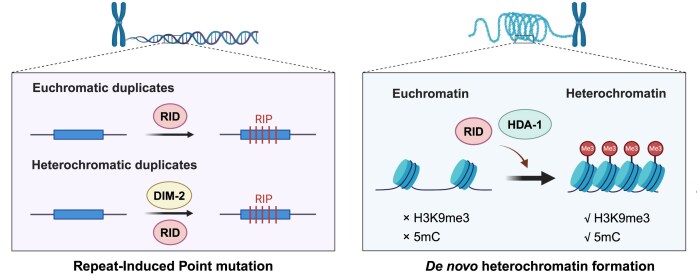

## Introduction

Eukaryotic genomes commonly have problems with repeat sequences, many of which are transposable element derived [1, 2]. A common response to these issues is to fold the potentially damaging sequence into largely inert heterochromatin, a highly condensed chromatin state, restricting their access to transcription and recombination [3]. Constitutive heterochromatin is often accompanied by repressive epigenetic modifications, such as histone H3 lysine 9 tail with 2 or 3 methyl groups (H3K9me2 or H3K9me3, depending on the organism) and DNA 5-methylcytosine (5mC) [4]. These modifications are catalyzed by histone-lysine N-methyltransferases (Suv39 class) and DNA methyltransferases (DMTs), respectively [5, 6]. Additionally, histone acetylation marks, which are associated with an active chromatin state, are removed by histone deacetylases (HDACs) [7, 8]. These modifications ensure that constitutive heterochromatin remains tightly folded and repressed in terms of expression, reducing the problem of “unwanted” transcripts [9]. The importance of such suppression is evidenced by reports of cancer when systems to trap “unwanted” transcripts are mutated [10, 11]. More generally, epigenetic aberrations are common in pathogenesis and are promising targets for therapies [12–14].

While the logic of forming heterochromatin over potentially harmful sequence for their inactivation is clear, the mechanistic basis is enigmatic. Indeed, how could a cell tell the difference between a DNA sequence that is needed and that which is not? Currently, the mechanisms underlying the recognition of repeat sequences, and the initiation of heterochromatin formation have yet to be fully elucidated [6, 15]. Currently, the best-known mechanism for recognizing repeat sequences is RNA interference through non-coding RNA (ncRNA). In addition to their post-transcriptional functions, small ncRNAs can bind to nascent transcripts and recruit repressors to promote heterochromatinization, thereby achieving silencing at the transcriptional level. In animals and plants, small interfering RNAs, PIWI-interacting RNAs (piRNAs), and various other ncRNAs have been reported to be involved in heterochromatin initiation [16–18].

The above mechanisms do not necessarily target duplicates but can do so if the RNA’s target sequences are shared between paralogs. However, there is also evidence that repeat sequences themselves may function as scaffolds to recruit downstream factors [19–21]. In this context, the fungus *Neurospora crassa* has long been used as a model organism for heterochromatin research and duplicate targeting [22–27]. In part, this is because, unlike in mammals in which methylation is essential, in *Neurospora* it not. More particularly, in addition to inactivation by heterochromatization, *N. crassa*, along with some of the Ascomycete fungi [28], also “attacks” repeat sequences via hyper-mutation, so-called repeat-induced point mutation (RIP). During sexual reproduction, in mitotic cell divisions immediately prior to meiosis, repeat sequences in the genome are recognized and trigger the RIP process resulting in a large number of C→T mutations [29, 30]. The presence of haploid genomes in individual nuclei most likely predisposes to such mutagenic activity as homology searching within a haploid nucleus enables targeting of paralogs exclusively [31–33].

Understanding the operation of RIP, and any coupling with heterochromatin formation, is thus potentially informative regarding the more general question, how does a cell distinguish repeat from non-repeat? In the genome of *N. crassa*, constitutive heterochromatin regions mostly colocalize with centromeric, pericentromeric and telomeric regions, in addition to other sporadic repeat sequences [23, 34]. Constitutive heterochromatin is defined by H3K9me3, which is catalyzed by DIM-5, a Suv39 class histone-lysine N-methyltransferase. Heterochromatin protein 1 (HP1) recognizes H3K9me3 and recruits the DMT DIM-2 to catalyze DNA methylation [35, 36]. *N. crassa* also has a further DMT homolog, RID (meaning RIp Defective), but this has not been functionally validated as a cytosine methyltransferase. Indeed, all known cytosine methylation in the asexual phase of *N. crassa* to date has been found to be catalyzed by DIM-2 [37–39]. Conversely, experiments using artificial stably integrated Repeat-Linker-Repeat (R-L-R) tester constructs report that RIP activity in duplicates is almost entirely halted in the absence of RID [25, 26, 38] suggesting a separation of roles for the two DMT homologs.

In this context, it was unexpected that our prior whole genome analysis of *N. crassa* reported appreciable amounts of RIP in *rid*^Δ^ strains [30]. This suggests that the prior model of RIP being exclusively mediated by RID is incorrect. What might we be missing? One possibility is that duplicates in the genomes are more physically distant than those in the R-L-R constructs. An alternative is that genetically more divergent repeats (i.e. many of the genomic ones) are handled differently. A further possibility is that old (most genomic duplicates) and new (e.g. R-L-R constructs) may be handled differently, because of their age, potentially assayed by chromatin status. Indeed, genomic duplicates also become marked with H3K9me3 histone marks and 5mC, leading to the complete silencing of the corresponding repeat elements or even functional genes [23]. This process is considered the main source of constitutive heterochromatin in the *N. crassa* genome but is not mechanistically well characterized [40–43]. Might it be that the RIP mechanisms are dependent on chromatin status and hence age?

As the DMT, DIM-2, mediates RID-independent mutation in single-copy regions adjacent to RIPed repeat sequences [26], rather than in the canonical duplicates in short repeat arrays, it is a candidate for enigmatic RIP-associated mutation. We thus seek to investigate whether DIM-2 and RID exhibit identical activity patterns in real genomes and tester experimental constructs.

We employ RID and DIM-2 homozygous knockouts (KO), plus the double KO, to ascertain the RIP rate for a variety of circumstances. We see that, unlike for the R-L-R constructs, for older repeats the double KO is needed to abolish RIP. This resolves the enigmatic behaviour of RIP in the genomic context and gives us a metric of RIP “types.” All tests point to age as the key variable explaining the R-L-R versus genomic difference. Employing R-L-R′ constructs, where R and R′ are divergent, we find that genetical divergence is not a key predictor, all such new constructs being RID dependent. To examine genomic distance, we compare RIP activity for old distant and old physically close repeats, finding that these have comparable RID/DIM-2 dependent profiles. We also utilize a pair of newly formed native duplicates showing these to have a characteristic “young” RIP phenotype comparable to R-L-Rs. These results strongly argue against extrapolation of experimental model systems (such as R-L-R) to genomic processes, the former eliciting the new duplicate responses, the latter mostly eliciting an old duplicate mode of response.

While RIP behavior depends on chromatin state, we also observe that RID can initiate heterochromatization. Using RID tethered to LexADBD, we demonstrate that RID can function as a nucleation center, initiating the transition in an HDA-1-dependent manner. Constitutive heterochromatin maintenance is however largely HDA-1 independent (HDA-1 paralogs are required). Thus, RID, primarily known for its role in triggering RIP, can play a role in *de novo* heterochromatin formation.

## Materials and methods

### *Neurospora* strains and culture conditions

*N. crassa* strains used in this study are listed in Supplementary Table S1. FGSC2489 was a gift from Chaoguang Tian, Tianjin Institute of Industrial Biotechnology, Chinese Academy of Sciences, China. FGSC3246 was purchased from Fungal Genetics Stock Center, Department of Plant Pathology, Kansas State University, USA. The *ku70^RIP^* strain was a gift from Qun He, College of Biological Sciences, China Agricultural University, China. WT strains with both AB copies were derived from the progeny of the cross between FGSC2489 and *ku70^RIP^*. The strains were grown, crossed, and maintained according to standard procedures [44], and the culture medium used in this research were based on the protocols offered by Fungal Genetics Stock Center (http://www.fgsc.net/) [45].

### Generation of the gene KO constructs

KO was carried out following standard pipelines [46]. Deletion cassettes were constructed with polymerase chain reaction (PCR) and all primers and synthetic oligonucleotides used in this study are listed in Supplementary Table S2. To generate the gene KO constructs, we amplified the regions upstream and downstream of the target gene using genomic DNA from strain *ku70^RIP^*. The *PtrpC::hph* resistance cassette, which confers resistance to Hygromycin [47], was amplified with plasmid pCSN44 as template. These three fragments were then combined by ‘stitching-PCR’ [48] and transformed into the strain containing *Sly1* AB or B by electroporation [49] to obtain gene KO strains. After screening for positive transformants with PCR, they were crossed with another mating strain to generate progeny.

### Generation of the R-L-R constructs

The DNA fragment containing 802 bp repeats (R) with complete or partial homology and a 729 bp linker (L) was synthesized and integrated into the *csr-1* locus by electroporation to get the R-L-R constructs. Inactivation of the *csr-1* gene served as a positive selectable marker [25, 50], and the positive transformants were verified by PCR and Sanger sequencing. This was similar to the test construct used by Gladyshev *et al.* [25, 26], while our 729 bp link is slightly longer. The repeat with 95% identity was PCR-amplified from RIPed sequences, which have a slightly increased AT ratio (AT% from 45.51% to 50.62%), while the 90% identity repeat was synthesized to maintain the same AT-ratio (AT% = 45.64%) in order to avoid potential DNA methylation.

### Generation of LexAO construct

The DNA fragment containing four copies of LexAO sequence was synthesized [43]. To insert the LexAO at an alternative euchromatic locus, *his-3*, we amplified fragments of the *his-3* gene's downstream region using strain *ku70^RIP^* genomic DNA as template, and the *PtrpC::hph* resistance cassette that was amplified as a selective marker. The region downstream of *his-3*, *PtrpC::hph* resistance cassette, LexAO sequence were combined by ‘stitching-PCR’ and transformed into strains containing B (*Sly1-1r*) or both of the A and B copies. The positive transformants were verified by PCR and Sanger sequencing.

### Construction of strains expressing LexADBD-tagged fusion protein

The DNA fragment containing 8 × glycine linker, the SV40 NLS, and the LexADBD sequence was synthesized [43, 51]. To express gene-of-interest fusions with this fragment, we amplified regions immediately upstream and downstream of the target protein's stop codon using genomic DNA from strain *ku70^RIP^* as template. The *PtrpC::bar* resistance cassette was synthesized as a selective marker. These four fragments were then combined by ‘stitching-PCR’ and integrated into strain containing *Sly1-1r* at the gene's original locus. LexADBD was expressed under the control of the target proteins’ endogenous promoter, and could be subject to gene expression discrepancies between hyphae and ascocarps. The positive transformants were verified by PCR and Sanger sequencing, the expression levels being confirmed using RT-qPCR.

### Genomic DNA extraction and whole genome resequencing

After growing on a plate for about 3 days at 32°C, the tissue was transferred into 2 mL microcentrifuge tubes and ground to a fine powder in liquid nitrogen. Subsequently, each sample was mixed with 0.8 mL lysis buffer (10mM Tris-HCl pH 8.0, 10mM EDTA pH 8.0, 0.1mM NaCl, 2% Triton-X100, 1% SDS) and incubated for 40 min at 65°C. DNA was extracted by phenol/chloroform/isoamyl alcohol method and re-sequenced individually. Whole genome resequencing was carried out at BGI Genomics. The data analysis was performed as described previously [30]. Each sample spore was sequenced to a depth of over 40-fold with more than 95% of the genome covered. In each of the sequenced samples, more than 90% of the reference genome could be covered with at least five reads.

### Identification of mutations

Mutations were called using similar methods to those reported before [30]. In brief, resequencing reads were mapped to the reference genome (version NC12, NCBI accession number GCF_000182925.2) [41] with BWA aligner [52]. Raw variants were called using HaplotypeCaller in Genome Analysis Toolkit [53].

Variants that passed the following filters were considered as candidate mutations: (i) For each variant site both parents and focal offspring should have a read-depth ≥ 5 with the mutated version of the allele present in offspring ascospores only, i.e. absent in parents. (ii) Due to the haploid nature of *Neurospora*’s nucleus, only variants with one type in a given sample were retained (i.e. not “heterozygous”), (iii) Variants should have a quality score >30, and be supported by reads on both plus and minus strands to avoid sequencing artifacts. Candidate mutations were further manually checked to exclude possible errors, including: (i) misalignment errors especially in polymer regions; (ii) variants called from error-prone mapping regions; (iii) variants present in parental samples that were not recognized by the variant caller.

### Detection and confirmation of the *Sly1* sequences

Most of the duplicates in the NC12 reference genome have been heavily RIPed, heterochromaticized and 5mC methylated. From the second generation cross progenies of FGSC2489 and *ku70^RIP^*, an 11-kb duplicated sequence that underwent heavy RIP was identified. Notably, this sequence exists as a single-copy in both parental strains, FGSC2489 and *ku70^RIP^*, where it is a single-copy unmethylated sequence on Chr06 native to strain FGSC2489 (named as copy A), and is a single-copy partially methylated sequence on Chr02 native to strain *ku70^RIP^* (named as copy B). Through literature searches and BLAST comparisons, it was determined that the copy in FGSC2489 had previously been confirmed as a DNA transposon, designated “*Sly1-1*” by Wang *et al.* [54].

To confirm the locations and sequences of *Sly1-1* copies, we carried out an assembly of the cross progeny (named “kuc4-1″) containing both A and B copies using Pacbio long-read sequencing. Alignment between NC12 reference and kuc4-1 genomes provided the accurate insertion locations of the A and B copies. WGS reads of strain *ku70^RIP^*, which only has copy B, and the FGSC2489 strain used in this work, which only has copy A, were mapped to the kuc4-1 genome, the single copy nature being further confirmed in the relevant strain. The meiotic recombinants with varying combinations of copy A and B allow us to measure RIP activity and heterochromatin status on native duplicates.

### Identification of mutations on *Sly1* sequences

To accurately identify mutations on *Sly1-1*, *de novo* assembly was used to reconstruct the RIPed sequence of progeny spores. Firstly, the WGS reads were mapped to NC12 reference genome [41] with BWA aligner [52] and reads mapping to *Sly1-1* were extracted from bam files using Samtools [55] and assembled with ABySS using default parameters [56]. The resulting scaffolds were then compared to the parental *Sly1-1* and *Sly1-1r* sequence and the mutations that arose during the cross were extracted.

### Definition of duplicate sequences

We utilized BLAST [57] to find duplicate sequences within the reference genome [58]. The reference genome was divided into 500bp non-overlapping windows, and windows overlapping with BLAST hits that had an identity greater than 65% were classified as duplicate (Dup) regions, while the others were classified as non-duplicate (non-Dup) regions.

To investigate the putative influence of physical distance, Dup regions were further categorized based on their relative locations (i.e. distances of each BLAST pair) on the chromosomes: less than 5 kb, between 5 and 300 kb, greater than 300 kb, and those on different chromosomes. Mutations were subsequently counted for all possible BLAST pairs and summarized according to these four distance categories.

### Bisulfite genomic sequencing and calculation of DNA methylation level

DNA libraries were prepared with Illumina TruSeq Methylation kit according to the manufacturer's instructions. Sequencing was performed on Illumina HiSeq 2500 system (BGI Genomics) to generate 150 bp paired-end reads. The methyl-seq data were processed using the Bismark tool [59]. The raw reads, were mapped to reference genome using Bowtie2 [60], and bismark_methylation_extractor was used to extract DNA methylation data of Cytosines. The methylation level of a specific window is calculated as DNA methylation level = methylated Cs / (methylated Cs + unmethylated Cs).

### Chromatin immunoprecipitation

All cultures were grown in Vogel's minimal medium with 2% glucose at room temperature for about 36 h. Subsequently, samples were crosslinked in 1% formaldehyde for 15 min and terminated by adding glycine. Samples were then collected by filtration, washed with phosphate-buffered saline (PBS), squeeze-dried, ground to fine powder in liquid nitrogen, suspended with lysis buffer (50 mM HEPES pH 7.5, 137 mM NaCl, 1 mM EDTA, 1% Triton X-100, 0.1% deoxycholate Na, 0.1% SDS) containing proteinase inhibitors, and sonicated to shear the chromatin. After sonication, samples were centrifuged at 10 000 rpm for 10 min to separate insoluble and soluble fractions. The resulting soluble fraction was then incubated with H3K9me3 antibody (39 062, Active Motif) overnight at 4°C under rotation. The following day, Protein A/G Magnetic Beads (HY-K0202, MCE) were added to each sample and incubated for 4 h at 4°C under rotation. After incubation, the beads were washed five times, and the bound proteins were eluted with elution buffer (1% SDS, 0.1M NaHCO_3_). Finally, elution and input fractions were decrosslinked at 65°C, DNA was then extracted by phenol/chloroform/isoamyl alcohol method. For quantitative ChIP, real-time PCR was performed using a StepOnePlus Real-Time PCR System (Applied Biosystems), and relative enrichment was determined by measuring enrichment as a percent of the total input.

### Cleavage under targets and tagmentation (CUT&Tag)

All cultures were grown on plate for about 3 days at 32°C and ground to fine powder in liquid nitrogen. The sample was then mixed with lysis buffer, which was supplemented with snailase and chitinase, and incubated for 2 h at 37°C. Subsequently, nuclei were extracted using the Nuclear Extraction Kit (SN0020, Solarbio), and CUT&Tag experiment was performed with the H3K9me3 antibody (39 062, Active Motif) according to the protocol provided by the Hyperactive Universal CUT&Tag Assay Kit (TD903, Vazyme). The enrichment of target sites in the library was detected by sequencing at Novogene using an Illumina HiSeq 2000 system.

### Bioinformatic analyses of ChIP-seq datasets

ChIP-seq and CUT&Tag raw read fastq files were mapped to the reference genome with bowtie2 [60], and output SAM files were transformed into bam files by samtools [55]. Deeptools [61] were used to produce bigwig files normalized by Reads per Kilobase Per Million Reads (RPKM). The Integrative Genomics Viewer [62] were used to create figures.

### Expression, extraction and purification of 6×his-tagged RID

To construct strains expressing 6 × His-tagged RID, we amplified the regions upstream and downstream of the stop codon of *rid* using genomic DNA from the *ku70^RIP^* strain. These regions were combined with 6 × His-tag and the PtrpC::*hph* resistance cassette as a selective marker. The resulting construct was then integrated into the native *rid* locus of *N. crassa*, ensuring that the 6 × His-tagged RID is expressed under the control of the native *rid* promoter. Total protein extracts were obtained by lysing sexual asci samples with lysis buffer (50 mM HEPES pH 7.4, 137 mM NaCl, 10% Glycerol) supplemented with protease inhibitors. The extracts were then centrifuged at 10 000 rpm for 10 min, and the supernatants were collected for purification using Ni-NTA Sefinose Resin. Subsequently, the 6 × His-tagged RID and its associated proteins were eluted with imidazole solution and separated by SDS–PAGE. The separated proteins were visualized using Coomassie blue staining and finally subjected to mass spectrometry for protein identification.

## Results

### Distinct roles of RID and DIM-2 in triggering RIP between native and artificially introduced duplicates

**Figure 1.**
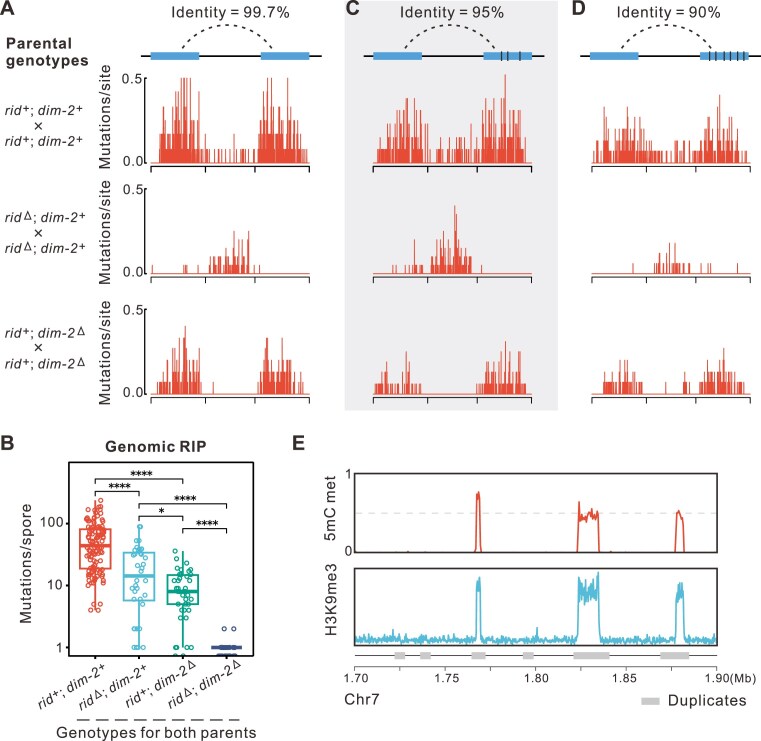
RIP patterns in R-L-R constructs and native RIPed duplicates colocalizing with heterochromatic regions. (**A**) RIP on R-L-R segments with different divergence. Besides the original R-L-R segment, two additional tester constructs with 5% and 10% divergence (**C** and **D**) were made in WT, *rid*^Δ^ and *dim-2*^Δ^ background. (**B**) The RIP on genomic duplicates from parents in WT, *rid*^Δ^, *dim-2*^Δ^ and *rid*^Δ^; *dim-2*^Δ^ background to progeny ascospores. Significance levels: ***P ≤ 0.001, **0.001 < *P* ≤ 0.01, *0.01 < *P* ≤ 0.05; ns, *P* > 0.05. (**E**) The colocalization of native duplicates, heterochromatic H3K9me3 regions, and 5mC methylation. 1.5–1.9 Mb on Chr7 is shown here.

To understand the roles of RID and DIM-2, we generated four crosses by mating parental strains deficient in either or both of the *rid* and *dim-2* genes, which were knocked out via homologous recombination (see Methods for details). We also incorporated a R-L-R tester construct, similar to that originally developed by Gladyshev *et al.* [25, 26], which retains repeat sequences identical to the previously employed construct but features a slightly longer linker sequence (Fig. 1A). A total of 54 tetrads, including 334 spores, from the four crosses were sequenced at a high coverage (>40-fold), enabling accurate detection of genomic mutations, including RIP events.

Comparing with wild type (WT), R-L-R constructs in the *dim-2*^Δ^ × *dim-2*^Δ^ background had a 50% reduction in mutation in the repeat elements (Fig. 1A). In *rid*^Δ^ × *rid*^Δ^ the rate was almost zero (Fig. 1A, Supplementary Table S3). In contrast to R-L-R constructs, the distribution of RIP at the native genomic duplicates appears to have distinct patterns when considering the influence of RID and DIM-2. First, disrupting either RID or DIM-2 alone does not eliminate most RIP activities in native genomic duplicates, a substantial number of mutations persisting in genomic duplicates of *rid*^Δ^ × *rid*^Δ^ or *dim-2*^Δ^ × *dim-2*^Δ^ crosses (Fig. 1B), exhibiting typical RIP characteristics, i.e. clustered G:C→A:T mutations. Knocking out RID alone only resulted in 67.9% reduction of RIP frequency (Fig. 1B, Supplementary Table S4), which is much lower than the 99.2% reduction observed in duplicates of R-L-R constructs (Fig. 1A, Supplementary Table S3). Knocking out DIM-2 alone has a large impact on RIP within native genomic duplicates, the RIP frequency being reduced by 81.8% (Fig. 1B). Only in *rid*^Δ^; *dim-2*^Δ^ double mutants, however, did the RIP activity became undetectable (Fig. 1B). Taken together, these results suggest that RID and DIM-2 likely have different contributions to RIP at the native genomic duplicates when compared with that in R-L-R constructs. In R-L-R, RID is key in genomic duplicates removal of both factors is needed to abolish RIP.

### Physical distance and sequence divergence between repeats do not determine RID/DIM-2 roles

Why might genomic duplicates and R-L-R constructs differ as regards the activity of RID and DIM-2? One possibility is that genomic duplicates are physically more distant i.e. not just separated by a short linker, a linker whose mutational rate is reduced to nearly zero in *dim-2*^Δ^ × *dim-2*^Δ^ (Fig. 1A, Supplementary Table S3). To assess the possible influence of physical distance, we extracted all possible pairs of duplicates present in the reference genome. A total of 5.84 Mb is defined as duplicate using a 65% identity cutoff [30, 39, 41], representing 14.4% of the genome. Duplicate pairs were categorized by their relative locations. Unlike artificial repeats (the R-L-R constructs), many genomic sequences that are identified as having at least one duplicate have multiple copies and may, as a consequence, be classified in multiple categories. We counted each instance. We found that most duplicates have corresponding pairs on different chromosomes, these making up 5.59 Mb of repeat sequences, while approximately 0.379, 1.98, and 3.12 Mb of genomic repeats are covered by duplicate pairs with a physical distance within 5kb, between 5 and 300 kb and over 300 kb, respectively (Supplementary Table S5). Comparison of mutation rates due to RIP prior to and post knocking out of RID or DIM-2 suggested no obvious influence of physical distance on the functions of both RID and DIM-2. Notably, for genomic duplicate pairs with a separation distance shorter than 5 kb—which may mimic the R-L-R structure—we observed reduced mutation rates in cross progenies of both *rid*^Δ^ and *dim-2*^Δ^ strains, indicating that these pairs do not have the profile of R-L-R constructs despite their genomic proximity (Supplementary Table S5). This indicates that the difference between genomic and R-L-R duplicates is not explained by the fact that most native genomic duplicates are not in close proximity.

Next, we ask whether sequence divergence between duplicates matters as R-L-R constructs employ identical repeats, but genomic duplicate pairs are normally more divergent. We designed R-L-R′ (5% divergence) and R-L-R″ (10% divergence) constructs, where the two repeats have a sequence identity of 95% and 90%, respectively (Fig. 1C and D). The two genetically more divergent repeats are treated the same as the R-L-R constructs by RID and DIM-2 (Fig. 1C and D), although the overall RIP efficiency slightly decreases when duplicates become more divergent (Fig. 1C and D). This further indicates that the difference between genomic and R-L-R duplicates also cannot be explained by the fact that most genomic duplicates are genetically more divergent.

### Roles of RID and DIM-2 are also different in mediating 5mC methylation in two sources of duplicates and are associated with historical 5mC methylation levels

If the difference in the relative importance of RID and DIM-2 on R-L-R and genomic duplicates is not owing to proximity or to genetic divergence, might chromatin status matter? DIM-2 is thought to be responsible for nearly all DNA methylation in vegetative growth of *N. crassa* [37, 38]. Knocking out of DIM-2 results in the complete elimination of DNA methylation in (non-artificial) genomic duplicates, whereas knocking out RID has no observable influence (Supplementary Fig. S1). For comparison, we knocked out DIM-2 and RID in WT strains with the R-L-R constructs, crossed them within the same genotypes, and measured the 5mC methylation levels in the hyphae of resultant cross progenies. Similar to its impact of non-artificial duplicates, disruption of DIM-2 again removes all RIP-associated DNA methylation in duplicates of R-L-R constructs (Supplementary Fig. S2). In contrast, unlike the rare impact of RID on 5mC methylation of non-artificial genomic duplicates (Supplementary Fig. S1), disruption of RID also removes nearly all 5mC methylation in duplicates of the R-L-R constructs (Supplementary Fig. S2). A slight difference between the impact of DIM-2 and RID on DNA methylation is observed in linker sequences, as DIM-2 is also necessary for the maintenance of 5mC methylation in linkers but RID seems to only affect part of the 5mC methylation there (Supplementary Fig. S2). This finding again confirms the distinct roles that RID and DIM-2 may play across different classes of sequence.

One of the intrinsic differences between genomic duplicates and artificially introduced duplicates is that the genomic duplicates normally have experienced multiple rounds of sexual cycles, so have been under the influence of historical RIP, while the artificially introduced duplicates have not. However, accurately determining whether a duplicate is historically RIPed or not is hard. Since in *N. crassa*, RIPed duplicates are typically cytosine methylated [63, 64] (Fig. 1E), we assessed whether the difference in historical 5mC methylation levels of two sources of duplicates influences the functions of RID and DIM-2.

Analysis of RIP rates as a function of regional methylation levels suggests that around 89.6% of RIP occurs in genomic duplicates where the DNA methylation level of corresponding regions in parental strains exceeds 10%, which constitutes 4.12Mb and 70.5% of all the duplicate regions (Supplementary Table S6). The mutation rate is significantly higher than that in the 1.72 Mb of duplicate sequence with methylation <10% (per sample per Mb mutation rate, *P*= 0.00095, Brunner–Munzel test), suggesting that the genomic RIP is disproportionately in established duplicates which have already been highly cytosine methylated prior to crossing. Comparatively, RIP in duplicates of artificially introduced R-L-R constructs normally exhibit no methylation in parental strains (Supplementary Fig. S2). These findings are consistent with the hypothesis that the historical DNA methylation status of duplicates (alongside other colocalized historical events like RIP and heterochromatin states) might have a role in promoting the distinct roles of RID and DIM-2 in triggering RIP.

### Confirmation of distinct roles of RID and DIM-2 using a newly formed pair of *Sly1* duplicates

**Figure 2.**
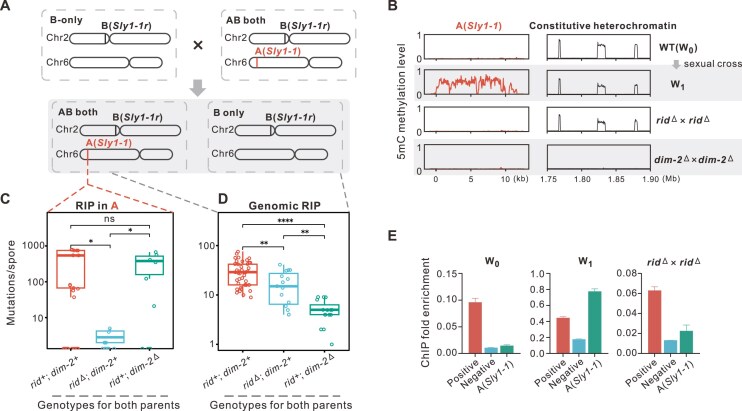
RID is tightly involved in RIP and *de novo* heterochromatin formation on unmethylated *Sly1-1*. (**A**) The hybridization diagram to investigate RID’s function on different types of duplicates. (**B**) RID controls *de novo* methylation on copy A (*Sly1-1*). The level of DNA methylation on *Sly1-1* was analyzed in WT, *rid*^Δ^ and *dim-2*^Δ^ backgrounds. Methylation level was calculated as 50bp windows for the 11 kb *Sly1-1* sequence and 500 bp windows for 1.75–1.9 Mb on Chr7 as the 5mC constitutive control. (**C**) and (**D**) RIP on *Sly1-1* and genomic duplicates from crosses of WT, *rid*^Δ^ and *dim-2*^Δ^ backgrounds to progeny ascospores. Significance levels: ***P ≤ 0.001, **0.001 < *P* ≤ 0.01, *0.01 < *P* ≤ 0.05; ns, *P* > 0.05. (**E**) H3K9me3 modification on *Sly1-1* requires RID. The relative enrichment of H3K9me3 at the site of *Sly1-1* was assessed by ChIP-qPCR in WT and *rid*^Δ^ background. Data were normalized to ratios obtained without immunoprecipitation (total input), and the H3K9me3 hotspot and coldspot as the positive control and negative control, respectively. Error bars represent the standard error of the mean.

Since artificially introduced constructs might accompany other unknown changes that trigger a different behavior of RID and DIM-2, ideally, we should confirm the above findings in the native genome without artificially introduced duplicates. We anticipate that if the sexual cycle-related historical events (marked by the changes in 5mC methylation, or RIP, or heterochromatin states) are involved in triggering the altered behaviors of RID and DIM-2 on duplicates, similar differences should also be observable between methylated and unmethylated duplicates in the native genome. As historical genomic duplicates are heavily methylated in common lab strains of *N. crassa*, we searched for recombinant spores from crosses of a range of strains and identified a strain with a pair of newly formed unmethylated duplicates (“Materials and methods”), a model for young duplicates that have not been artificially introduced. The two copies of this duplicate pair are located on different chromosomes and are confirmed to be previously described DNA transposons [54]. The two copies are hereafter named *Sly1-1* (or copy A for simplicity when describing crossing experiments) and *Sly1-1r* (or copy B) (Fig. 2A, Supplementary Figs S3 and S4). Their location and sequence were further confirmed by a bespoke long-read assembly method that distinguished between the copies (“Materials and methods,” Supplementary Fig. S3). The identity of two copies is 97.4%. *Sly1-1r* contains 309 G:C-A:T differences compared to *Sly1-1*. The length of each copy is ∼11 kb (Supplementary Fig. S4). This length and identity should be (and is) sufficient to trigger RIP in subsequent crossing experiments.

Copy A does indeed resemble one repeat of an R-L-R like externally introduced duplicate in that its DNA sequence is unmethylated. In WT parental strains, 5mC methylation of the A copy is nearly undetectable initially (Fig. 2B). We conducted crosses between strains containing both A and B copies and strains with the B copy alone, before and after knocking out either RID or DIM-2, to investigate the roles of these proteins on duplicates of different 5mC methylation status (Fig. 2A and Supplementary Table S7). Crossing WT AB and B-containing strains (WT^AB^ and WT^B^) results in massive RIP (211.2 mutations per spore in the A copy, Fig. 2C, Supplementary Table S7) with significantly increased methylation levels (33%) in the A copy (WT first generation progenies, W_1_ in Fig. 2B). The large variance of mutation rates can be attributed to approximately half of spores being without RIP in the A copy, mirroring the ∼50% RIP frequency previously reported in unlinked-duplicates [65]. When crossing between AB-*rid*^Δ^ strains and B-*rid*^Δ^ strains, almost no RIP, DNA methylation, or H3K9me3 is observed in the A copy (Fig. 2B, C, and E), while 20.9 RIP-dependent mutations remain present in other historically methylated genomic duplicates (Fig. 2D). This mirrors the central role of RID in RIPing (young) R-L-R constructs (Fig. 1A). Conversely, in the *dim-2*^Δ^ × *dim-2*^Δ^ background, RIP activity was less affected in the A copy when compared to other genomic duplicates (Fig. 2C and D) while 5mC methylation is absent, as also observed for constitutive heterochromatin (Fig. 2B). This too mirrors the lesser importance of DIM-2 on R-L-R constructs as regards RIP.

These results also suggest that the historical 5mC methylation status of duplicates is likely to be involved in promoting the distinct functional roles of RID and DIM-2, where RID acts as a primary facilitator of RIP in cytosine unmethylated duplicates, while both RID and DIM-2 are required to mediate RIP in historically methylated duplicates. Perhaps most intriguing is that RID alone is capable of triggering *de novo* 5mC methylation in unmethylated duplicates, while such capability is not observed for DIM-2.

### RID has a role in promoting *de novo* heterochromatin formation

Besides being frequently methylated, the RIPed duplicates are generally organized in the form of constitutive heterochromatin marked by H3K9me3 [39, 42, 63, 64, 66] (Fig. 1E). Previous research suggests that A:T-rich DNA, produced by RIP, may act as recruitment signal for heterochromatin formation [42, 64, 67, 68]. Given the finding that RID can trigger *de novo* 5mC methylation in euchromatic unmethylated duplicates, we sought to test whether RID can promote heterochromatin formation without the involvement of RIP.

**Figure 3.**
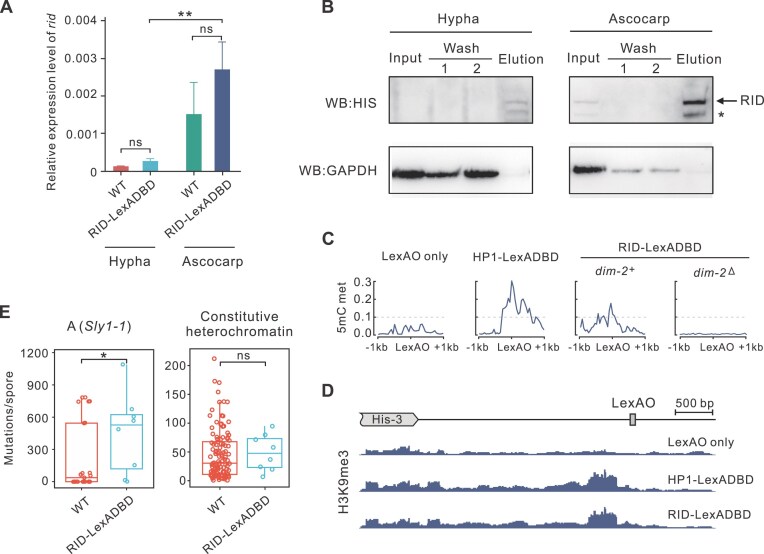
RID-LexADBD under native *rid* promoter could induce RIP and *de novo* heterochromatin formation. (**A**) Quantification of *rid* expression levels in asexual hypha and sexual ascocarp using RT-qPCR, including both the WT and RID::LexADBD strains. (**B**) Western Blot analysis of 6xHis::RID in hypha and ascocarp shows much higher expression in the sexual stage, while expression in asexual stage is barely detectable. (**C**) Tethered RID induces DNA methylation at a euchromatic locus. The LexADBD-fused protein was targeted to LexAO sequence integrated downstream of the *his-3* locus. Before any protein is tethered, LexAO as well as surrounding regions are confirmed to be unmethylated (methylation level in 50 bp windows). When HP1 or RID was tethered in vegetative hyphae, DNA methylation was induced only when *dim-2* is present. (**D**) Tethered RID can establish H3K9me3 near the region of LexAO. Enrichment of H3K9me3 is displayed using the Integrative Genomics Viewer, and the *y*-axis represents RPKM. (**E**) The rate of mutation occurring on *Sly1-1* (left) and constitutive heterochromatin (right) were compared between WT and RID-LexADBD backgrounds. The mutation rate in RID-LexADBD is significantly higher for *Sly1-1* (Brunner–Munzel test, *P* = 0.022 for *Sly1-1* and *P*= 0.48 for constitutive heterochromatin).

To elucidate RID’s role in heterochromatin formation, we employed a LexA-based *in vivo* protein-tethering system [43]. This system employs the LexA DNA-binding domain (LexADBD) fused with the target protein of interest and an integrated LexA consensus sequence (4 × LexAO) that recruits the fused protein leading to ectopic DNA binding. We employed RID fused with LexADBD under control of the *rid* promoter. The 4 × LexAO sequence was located downstream of the euchromatic *his-3* locus. The fused RID-LexADBD has an expression pattern similar to WT (Fig. 3A and B). The LexAO locus and surrounding regions are cytosine unmethylated and without H3K9me3 peaks prior to tethering (Fig. 3C and D). In the RID-LexADBD system, we observed a higher RIP rate in *Sly1-1* compared to WT (left in Fig. 3E, Brunner–Munzel test, *P* = 0.022), while the RIP rate in normal constitutive heterochromatin remained similar between RID-LexADBD and WT (right in Fig. 3E, Brunner–Munzel test, *P* = 0.48), confirming that the modified RID retains its canonical function in RIP. Unlike DIM-2, which does not induce DNA methylation at the LexAO locus [43], tethered RID was able to induce both H3K9me3 and DNA methylation at this locus (Fig. 3C and D). This suggests that RID can induce H3K9me3 and 5mC methylation prior to the initiation of RIP, suggesting a role for RID in *de novo* heterochromatin formation in euchromatic regions.

To ask whether RID could induce DNA methylation independent of DIM-2, we measured the 5mC level on LexAO in a RID-LexADBD *dim-2*^Δ^ background. We found that there is no detectable 5mC level when DIM-2 is disrupted (Fig. 3C), suggesting that RID could induce DNA methylation but only in a DIM-2 dependent manner.

### RID can induce mutations in non-duplicates

The variable influences of RID on duplicates as a function of the age/methylation raises an interesting question as to whether RID might also influence single copy genes/regions. For example, can RID induce RIP-like mutations in a single-copy region? The above constructed RID-LexADBD strains are appropriate to test this possibility. A control system with only the LexAO segment but lacking RID in the LexADBD setup was also constructed to rule out the influence of tethering itself. Both the strains with RID tethered to the single-copy LexAO region and the control strains were crossed, and the resultant spores were sequenced to assess whether it can still trigger many mutations, mimicking RIP.

For parental strains (i.e. in an asexual phase), Sanger sequencing confirmed no mutations were induced within the 1000 bp single-copy LexAO region with or without tethered RID (Supplementary Fig. S5). This suggests that although directly tethering RID to LexAO region can trigger *de novo* 5mC methylation and heterochromatin, it failed to produce any mutations there during the asexual phase. After crossing (i.e. having undergone the sexual stage), we observed a total of 20 independent base-substitution mutations across 92 progeny individuals (Supplementary Fig. S5) from the RID-tethered system, yielding a mutation rate of 2.2*10^−4^ per site per sexual generation. In contrast, no base-substitution mutations were detected among 76 progeny individuals from the control system. This shows that, in contrast to the results in asexual phase, a high mutation rate, rivaling classical RIP levels, can be achieved once entering the sexual phase even in non-duplicated regions. One interpretation of these findings is that induction of RIP not only requires direct recruitment of RID, but also requires collaboration of other factors which are likely to only be available in the sexual stage. A further possible reason, however, is that, though detectable, the expression level of *rid* is much lower in hypha than in sexual ascocarps (Fig. 3A and B), which might hamper the capability of tethered-RID to carry out further processes in the asexual stage.

### RID-mediated heterochromatin formation requires HDA-1

To investigate the factors mediating RID-dependent heterochromatin formation, we constructed a strain expressing RID-his fusion protein and purified tagged RID for protein identification via mass spectrometry (See Methods for details, Supplementary Table S8). The identified peptides include three HDACs, HDA-1, HDA-2 and HDA-3, known to be crucial for heterochromatin formation in multiple organisms [36, 69–71]. These HDACs catalyze the removal of acetyl groups on lysine tails of histones, which are thought to be active histone marks. To confirm their roles related to heterochromatin, we generated KO mutants for each HDAC and evaluated their impact on constitutive heterochromatic regions as well as unmethylated euchromatic regions (e.g. *Sly1-1* segment).

**Figure 4.**
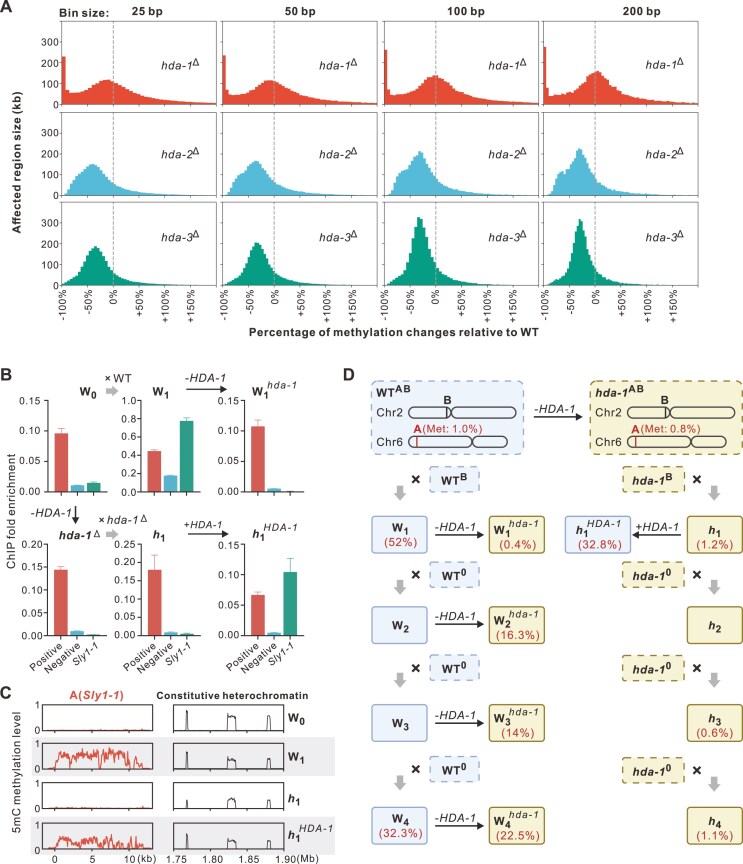
HDA-1 is required for RID-mediated *de novo* heterochromatin formation. (**A**) Distinct influence of HDA-1 and HDA-2/3 on maintenance of 5mC methylation. The genome was divided into 100 bp windows, and the methylation level for each window was quantified as 5mCs/(Cs + 5mCs). The histogram shows regions with altered methylation levels compared to WT, calculated as (“methylation level of mutant”—“methylation level of WT”)/ “methylation level of WT,” where 0 indicates no change and −100% indicates complete loss. Different bin sizes were tested, and only bins with a methylation level > 0.1 in WT were included to highlight the influences of HDACs on methylation maintenance. (**B**) HDA-1 is necessary for H3K9me3 induction on *Sly1-1*. The relative enrichment of H3K9me3 at the site of *Sly1-1* was assessed by ChIP-qPCR in WT, *hda-1* knocked out and re-introducing strains. Data were normalized to ratios obtained without immunoprecipitation (total input), and the H3K9me3 hotspot and coldspot as the positive control and negative control, respectively. Error bars represent the standard error of the mean. (**C**) 5mC Methylation changes on *Sly1-1* and constitutive heterochromatin after HDA-1 KO and its re-introduction. Methylation levels are quantified in 50 bp windows for the 11kb *Sly1-1* sequence (left) and in 500 bp windows for 1.75–1.9 Mb on Chr7, the 5mC constitutive control (right). The 5mC methylation level of WT parent (W_0_), the sexual progenies of WT (W_1_), sexual progenies of *hda1*^Δ^ (*h*_1_) and *h*_1_ with reintroduced HDA-1 (*h*_1_^HDA-1^) were shown. KO of HDA-1 hindered the increase of methylation level on *Sly1-1*(**A**) but do not affect the methylation level at many constitutive heterochromatic regions. (**D**) HDA-1 is essential for maintenance of methylation in transitional heterochromatin but is dispensable for stable constitutive heterochromatin. Strains containing AB copies were repeatedly crossed in both *hda-1*^+^ and *hda-1*^−^ background and DNA methylation levels were assessed in both WT and knock out strains. The percentages represent the level of DNA methylation of *Sly1-1*(**A**) before and after HDA-1 KO.

KO experiments revealed that a few (13.5%) methylated regions are dependent on HDA-1 in a highly localized (i.e. locus-specific) manner, where disrupting HDA-1 leads to striking 5mC methylation loss (Fig. 4A, Supplementary Figs S6 and S7), consistent with prior findings [70, 71]. For the majority of the genome, however, the mean cytosine methylation is unchanged (note unchanged peak of distribution in top panel of Fig. 4A and Supplementary Table S9). This pattern of variation is largely maintained over multiple generations (Supplementary Fig. S8). Meanwhile, disrupting either HDA-2 or HDA-3 leads to a more modest but uniform reduction in DNA methylation levels (note shift of peaks of lower two panels in Fig. 4A). The differing profiles of methylation level changes, with *hda-1*^Δ^ having rare but complete loss and *hda-2*^Δ^/*hda-3*^Δ^ having broader scale but modest reduction, suggest divergent roles of these three HDACs in the maintenance of DNA methylation of different loci, with HDA-1 distinct from HDA-2 and 3.

The methylation of recent duplicates that we identified are affected profoundly by HDA-1. Disrupting HDA-1 in parental samples (*hda-1*^Δ^) completely hindered the establishment of both H3K9me3 and DNA methylation in *Sly1-1* sequence among the ascospores (referred to as *h*_1_) derived by sexual process (Fig. 4B and C). In contrast, the ascospores from *hda-2*^Δ^ or *hda-3*^Δ^ mutants still exhibited moderate DNA methylation levels (average at 14% and 10%) (Supplementary Fig. S9). Reintroducing HDA-1 by transformation of the original gene segment in h_1_ spores restored the H3K9me3 and 5mC DNA methylation level on *Sly1-1* (*h*_1_^HDA-1^, Fig. 4B and C). These findings indicate that HDA-1 is crucial for the RID-dependent *de novo* establishment of heterochromatin during the sexual cycle but may be dispensable for the maintenance of H3K9me3 and 5mC methylation at many constitutive heterochromatic regions.

We also investigated whether HDA-1 is involved in the RID-dependent RIP. Among 17 ascospores from the cross of *hda-1*^Δ^ × *hda-1*^Δ^, we detected no difference in RIP mutation rates between strains with or without HDA-1 (Supplementary Fig. S10, *P* = 0.99 for *Sly1-1* and *P* = 0.24 for *Sly1-1r*, Brunner–Munzel tests), suggesting that RID-dependent RIP activity is independent of HDA-1.

To further validate the functions of HDA-1, we performed a progressive heterochromatin formation experiment by repeatedly crossing ascospores with both AB copies with a strain containing neither the A nor the B copy (referred to as “0”-containing strains) for three subsequent generations (progenies of WT × WT and *hda1*^Δ^ × *hda1*^Δ^, 1st–4th generation, abbreviation as W_1_ to W_4_ and *h*_1_ to *h*_4,_ Fig. 4D). This procedure generated at least three chromatin states in the A copy, including euchromatin (parental samples), transitional *de novo* heterochromatin (W_1_, first generation), and stable constitutive chromatin (W_2_ to W_4_, 2nd–4th generation). We also manipulated HDA-1 and RID in different generations to verify their impacts. By measuring the changes in each generation, our results revealed that the influence of HDA-1 decreases with each crossing. In W_2_, the cytosine methylation level remains high after KO (16.3%), which was much higher than that in first generation *hda-1* mutants W_1_*^hda-1^* (*P* << 0.001, Brunner–Munzel Test), indicating a chromatin state independent on HDA-1, different from the transitional heterochromatin in W_1_. Eventually in W_4_, the methylation level reached 32.3% and 22.5% before and after knocking out of HDA-1. The cytosine methylation level in the KO mutant is still significantly lower than in that in W_4_ (*P* << 0.001, Brunner–Munzel Test) but was much higher than that in KO mutants of W_1_*^hda-1^* to W_3_*^hda-1^* (0.4%, 16.3%, and 14.0%, *P* <<0.001 for all comparisons, Brunner–Munzel Test). Meanwhile, repeated crossing of AB-containing and “0”-containing strains in *hda-1*^Δ^ background (*h*_1_ to *h*_4_) failed to induce DNA methylation even in the fourth generation (1.1% in *h*_4_), despite multiple turns of RIP raising the AT ratio of the A copy from 48% to 61%. These results confirmed that the function of HDA-1 is necessary for *de novo* establishment of 5mC methylation during the early stage (W_1_) but becomes dispensable once heterochromatin is stably established into constitutive heterochromatins (W_2_ to W_4_).

**Figure 5.**
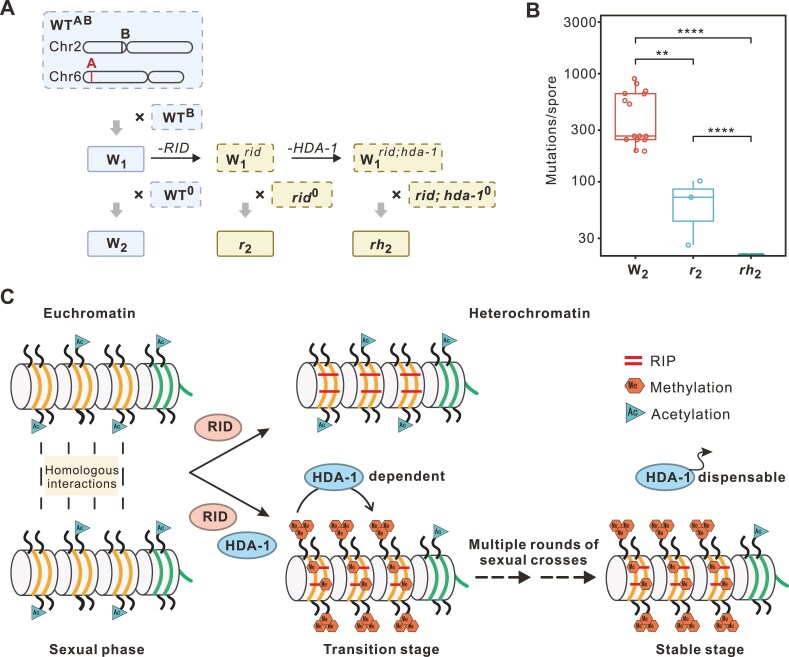
Transition from euchromatin to constitutive heterochromatin in *N. crassa*. (**A**) Schematic of the RID and HDA-1 KO in different generations. (**B**) RID functions differently in RIP on *Sly1-1*(**A**) from W_1_ to W_2_. KO of RID results in complete loss of RIP in first-generation progeny ascospores (Fig. 2C) but a few RIP mutations remain in second-generation ascospores (r_2_). The remaining few mutations disappear after additional KO of HDA-1. Error bars represent the standard error of the mean (s.e.m.), and significance levels are indicated as follows: ***P ≤ 0.001, **0.001 < *P* ≤ 0.01, *0.01 <*P* ≤ 0.05; ns, *P* > 0.05. (**C**) Model for *de novo* heterochromatin formation of unRIPed duplicates. DNA is shown wrapped around nucleosomes with a region of repeats, and the dashed lines represent the repeated DNA interactions in the sexual phase. In the upper section, RIP (horizontal bars) can be mediated by RID. In the lower section, RID and HDA-1 are crucial for the establishment of DNA methylation and H3K9me3, serving as signals for the formation of *de novo* heterochromatin. Subsequently, the transition of *de novo* heterochromatin into constitutive heterochromatin is dependent on HDA-1. After multiple rounds of RIP, constitutive heterochromatin becomes insensitive to HDA-1.

We also assessed the impact of knocking out RID or HDA-1 in intermediate generations on RIP activity, comparing it to that in the first generation ascospores (Fig. 5A). In euchromatic *Sly1-1*, knocking out RID essentially ceases all RIP activity (Fig. 2C). However, if RID is knocked-out in the W_1_ (W_1_*^rid^*), a low-level RIP activity in *Sly1-1* remains detectable in progeny ascospores (*r*_2_, Fig. 5B). This again confirms that RIP in established heterochromatin is governed by both RID and other factors (DIM-2), while in young unmethylated duplicates it is more RID dependent. Furthermore, inactivation of both RID and HDA-1 (W_1_*^rid;hda-1^*) abolishes all RIP activity in *Sly1-1* of the progenies (*rh*_2_, Fig. 5B), mimicking the *rid*^Δ^; *dim-2*^Δ^ double mutants. This implies that HDA-1-dependent heterochromatin is essential for RID-independent (e.g. DIM-2-mediated) RIP processes.

Considering the importance of HDA-1 in the RID-dependent *de novo* heterochromatin formation, we also asked whether the two proteins function interactively. To test whether HDA-1 directly binds to RID, we carried out yeast 2-hybrid assays but failed to detect robust interaction (Supplementary Fig. S11). Considering the weak peptide signals in mass spectrometry of HDA-1 as well as the knock out results (Fig. 4), it is more likely that HDA-1 and RID have either indirect interactions or that their interactions require other uninvestigated factors. Taken together, these results indicate that HDA-1 is required in the RID-dependent *de novo* establishment of heterochromatin, but is not involved in the RID-dependent RIP process.

## Discussion

How can it be that, in the absence of RID, no RIP is seen in R-L-R constructs, whilst RIP remains in genome-wide duplicates? We find that the DMT, DIM-2 is also required for RIP in this latter circumstance. The transition from RID alone to RID/DIM-2 dependent RIP we show to be a function of age, mediated by chromatin status, rather than either physical proximity or sequence divergence. In euchromatic duplicates, the RIP process affecting repeats relies solely on RID (DIM-2 modulates mutation rates in the intervening sequence but not otherwise). In contrast, in heterochromatic duplicates, both RID and DIM-2 are needed for the RIP process, though each contributes fewer mutations independently. This adds to mechanisms by which chromatin and mutation are coupled [25, 26, 72], notably, H3K9me3 and 5mC modifications can affect mutation rates directly or indirectly [25, 26, 39].

Our results also present a cautionary story as the differences between mechanisms of mutation associated with older and younger duplicates, argue against extrapolation of experimental results exclusively modeled using newly introduced duplicates (R-L-R constructs) to understand the mechanisms involved in those duplicates that have a more extensive history of sexual passage. In this instance, genome scale mechanisms likely differ for the most part from model experimental systems.

### *De novo* heterochromatin formation mediated by RID

RID, we have discovered, functions not only in the mutation process but also in the transition to heterochromatinization process. During sexual reproduction of *N. crassa*, once two repetitive sequences are recognized, RID is recruited to mediate RIP mutations in the region, alongside the establishment of heterochromatin marked by H3K9me3 and 5mC methylation, in which other repressing factors like HP1 and DIM-2 may also be involved. Further experimentation using a LexA-based *in vivo* protein-tethering system demonstrated that tethered RID can induce the formation of H3K9me3 and DNA methylation markers at a euchromatic single-copy locus during the asexual stage, without detectable RIP mutations. RID’s ability to initiate local DNA methylation requires DIM-2, supporting the understanding that, while a homolog of DMTs, RID appears not to have cytosine methylation ability. In contrast to the activity during the asexual phase, when this system undergoes sexual reproduction, tethered RID induces both RIP mutations and the establishment of H3K9me3 and DNA methylation marks.

These findings are consistent with a model in which RID has the potential to mediate *de novo* heterochromatin formation, largely independent of RIP mutations. The formation of *de novo* heterochromatin mediated by RID does not depend on the sexual reproduction process, whereas the RIP mutations mediated by RID do. This process occurs independently of DNA methylation or RIP mutations, highlighting the dual functionalities of RID and provides a pathway for chromatin state transition, emphasizing RID’s role in protecting the genome against repetitive sequences. The relevance of these results from an artificial construct for activity during the asexual phase come with the caveat that the expression level of RID in the asexual phase is low.

Strategically that RID has dual roles in RIP and heterochromatization makes sense in that the two are coupled processes. If duplicates in the genome are unwanted transposable elements, then mutation (RIP) and heterochromatization, should both be part of the same inactivation and degradation strategy. As RID is a member of the ancient DMT family our results hint at the possibility that it may have been ancestrally involved in the eu- to hetero- chromatin transition via H3K9me3 and DNA methylation, that was subsequently recruited to a repeat-specific mutational process. When it lost its DNA methylation functionality is unresolved.

The discovery of RID’s role in transitional heterochromatin assembly is perhaps most notable in that, while we know of the importance of chromatin marks for gene silencing and heterochromain formation [4, 15, 36, 73, 74] and several other factors are also known to facilitate the assembly of heterochromatic structures, including Clr4/Suv39h and HP1/Swi6 [75–78], the mechanisms guiding the recruitment of these factors to nucleation sites remain poorly understood. RID would appear to be a newly identified recruitment factor.

### Transition to constitutive heterochromatin requires HDAC1

Our results also indicate a division of labour between HDACs. The establishment of constitutive heterochromatin requires the removal of acetylation modifications indicative of active chromatin, a process catalyzed by HDACs [69, 71, 77, 79]. HDACs play crucial roles in heterochromatin establishment and maintenance. In *N. crassa*, there are four HDACs, HDA-1∼HDA-4, with HDA-1 being reported to be involved in constitutive heterochromatin formation [36, 43, 70, 71]. Our results suggest that HDA-1 is involved in the transition to heterochromatin, but not so much its maintenance. Our young duplicate's methylation was prevented on HDA-1 KO but the same KO otherwise leads to very highly localized loss of methylation leaving most heterochromatin untouched. Accordingly, as the young duplicate becomes old, by being passed through sexual cycles, HDA-1′s influence wanes. Ectopic heterochromatin establishment, facilitated by tethering heterochromatin factors or LSD1 KO [24, 43], also depends on HDA-1.

In our study, we observed that after RIP process, a signal for subsequent heterochromatinization is established on the duplicates, which become AT-rich (possibly itself associated with the heterochromatization). During this transition stage, downstream H3K9me3 and DNA 5mC modifications require HDA-1. In *hda-1*-deficient background, heterochromatin marks are absent on the duplicates in first generation progenies (h_1_), even with RIP mutations intact. Reintroduction of HDA-1 restores the 5mC levels to as high as those in WT W_1_ hyphae, while the KO of HDA-1 in WT W_1_ erases most heterochromatin marks, indicating that both heterochromatin establishment and maintenance (on young duplicates) strongly depend on HDA-1.

We also observed a conversion from a transitional state to constitutive heterochromatin, with the former sensitive to *hda-1* KO while only a small portion of the latter is dependent on HDA-1. After multiple crosses and RIP events, the heterochromatin state on the duplicates in the fourth generation (W_4_, Fig. 4D) becomes recalcitrant to *hda-1* KO. This conversion does not occur in an *hda-1*-deficient background, even after multiple RIP cycles, suggesting a dependency on HDA-1. In conclusion, HDA-1 is crucial for both *de novo* heterochromatin establishment and the conversion from a transition state to constitutive heterochromatin (Fig. 5C).

The initiation role for HDA-1 contrasts with the roles of HDA-2/3. Strains bearing deletions of the genes encoding these HDACs do not have an effect on the young duplicates but reveal global methylation reduction of heterochromatin (but not full loss). We note that while our HDA-1 results accord with previously described locus specificity [70, 71], our results for HDA-2 and HDA-3 contrast with prior Southern based analyses in which KO of *hda-2* and *hda-3* were argued to have no effect [71]. We suggest that the discrepancy may be accounted for by the semi-quantitative nature of the Southern approach previously employed that is prone to missing modest effects [80, 81]. Whether HDA-1′s role in the *de novo* establishment of heterochromatin on repeat sequences reflects an evolutionarily conserved functionality, or a lineage-specific oddity, remains to be seen.

## Supplementary Material

gkaf263_Supplemental_File

## Data Availability

The complete DNA sequencing, 5mC methylation sequencing, and ChIP-seq data generated during this study are available in the National Center for Biotechnology Information Sequence Read Archive repository under study accession number PRJNA1165970. The mass spectrometry data have been deposited in PRIDE (https://www.ebi.ac.uk/pride/) under project PXD060025. Sequences of the R-L-R constructs, *Sly1-1* and *Sly1-1r*, positions of duplication regions, and custom codes used in this study are deposited in https://github.com/jujushen/NcrassaRID and https://doi.org/10.5281/zenodo.15057073.
