# Supplementary material for: RID is required for both repeat-induced point mutation and nucleation of a novel transitional heterochromatic state for euchromatic repeats
Source: Nucleic Acids Res. 2025 Apr 4;53(6):gkaf263. doi: 10.1093/nar/gkaf263 (PMC11969663; doi:10.1093/nar/gkaf263)
Supplement: gkaf263_Supplemental_File [file gkaf263_supplemental_file.pdf]

Supplementary Information

RID is required for both Repeat Induced Point mutation and nucleation of a novel transitional heterochromatic state for euchromatic repeats

Zhen He, Nannan Wu, Ruonan Yao, Huawei Tan, Yingying Sun, Jingxuan Chen, Lan Xue, Xiaonan Chen, Sihai Yang, Laurence D. Hurst, Long Wang, Ju Huang

Supplementary Figures

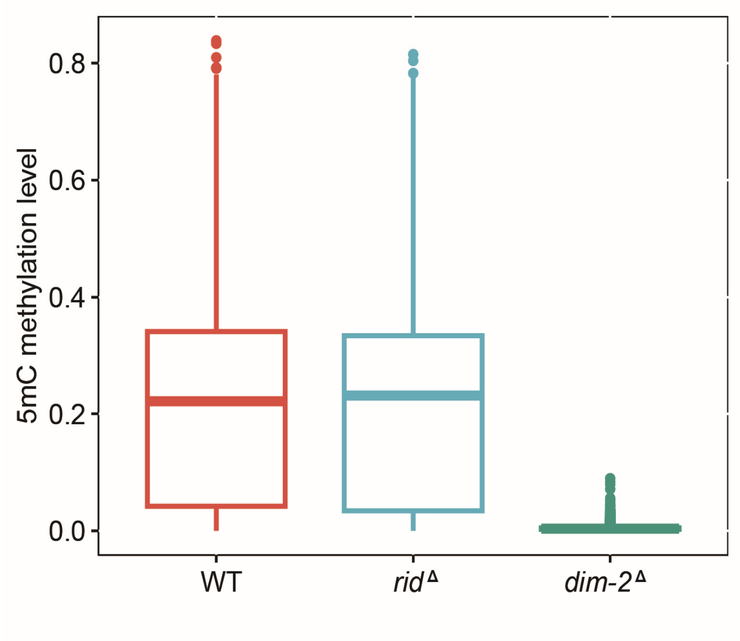

Supplementary Figure S1. DNA methylation level of genomic duplicates in wild-type, *rid*$^{\Delta}$ and *dim-2*$^{\Delta}$. The level of 5mC methylation in the genomic duplicates was measured in wild-type, *rid*$^{\Delta}$ and *dim-2*$^{\Delta}$ strains (quantified in 500 bp windows).

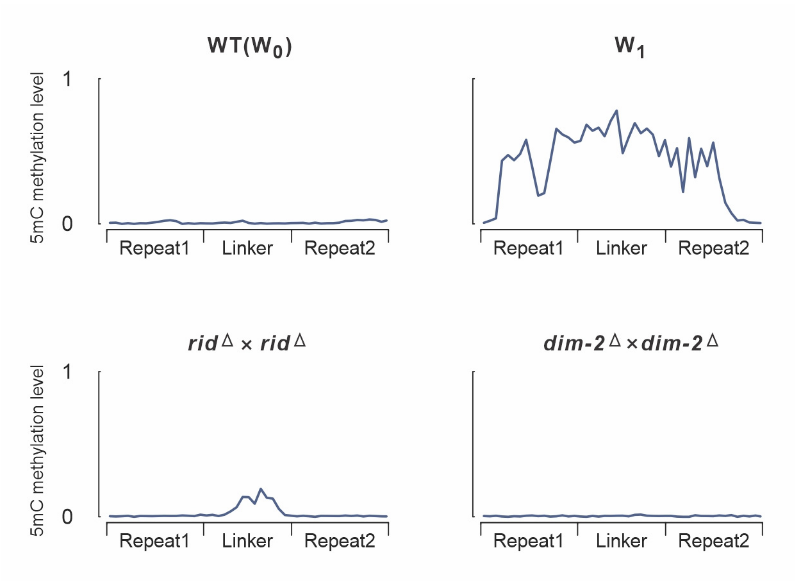

Supplementary Figure S2. Cytosine methylation distribution of the R-L-R constructs. Top row (left to right): W_0, wild-type parent; W_1, 1st generation progeny of wild-type; bottom row (left to right): 1st generation progeny of *rid*$^{\Delta}$ × *rid*$^{\Delta}$ and *dim-2*$^{\Delta}$ × *dim-2*$^{\Delta}$, respectively. The 5mC methylation level was quantified in 50 bp windows.

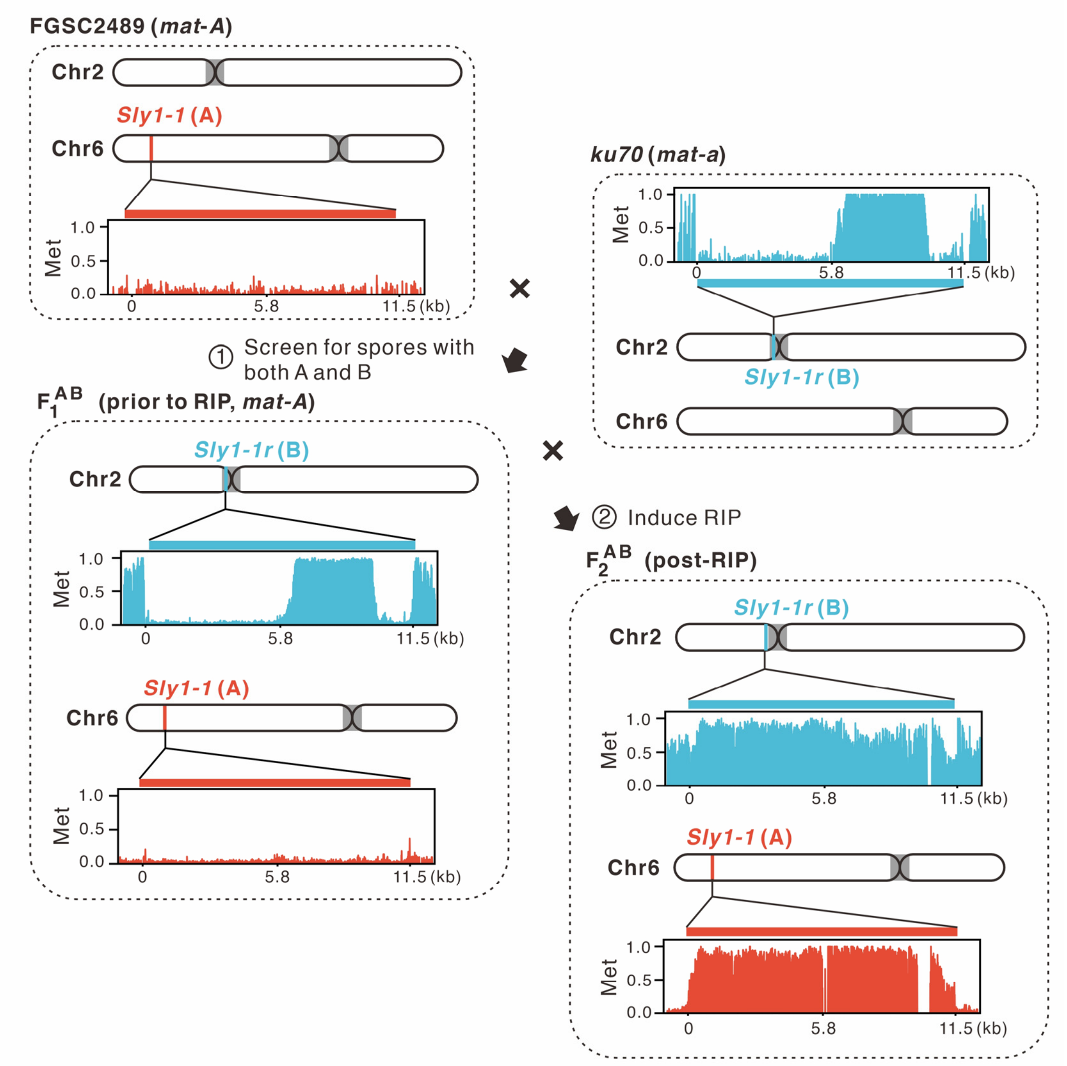

Supplementary Figure S3. Schematic illustration of the construction of a pair of young native duplicates. Relative methylation levels of each duplicate (pre- and post-cross) are represented in rectangles, with red indicating *Sly1-1* (or A) and blue indicating *Sly1-1r* (or B). Centromeric regions are marked with grey.

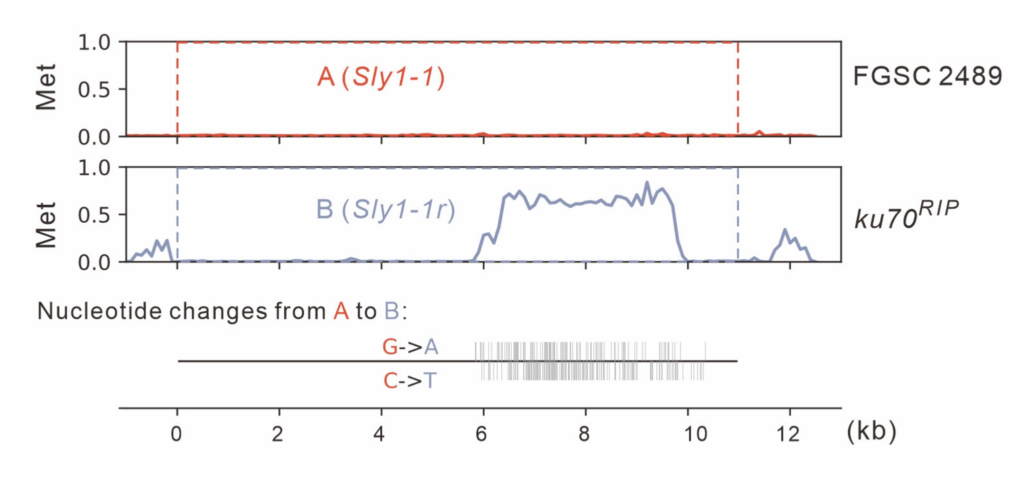

Supplementary Figure S4. Comparison of 5mC methylation levels and nucleotide differences between *Sly1-1* and *Sly1-1r* in parental strains FGSC 2489 and *ku70*^{RIP}. Methylation levels are quantified using 50 bp windows. Nucleotide difference between *Sly1-1* and *Sly1-1r* are indicated with grey bars, while the remaining regions are identical between the two copies.

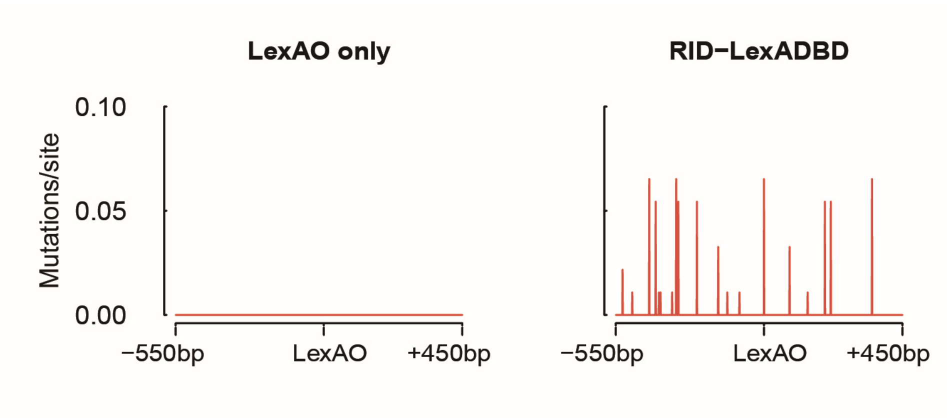

Supplementary Figure S5. Mutations of the region around LexAO. Mutation profiles of the region around LexAO in the crosses of LexAO only and RID-lexADBD. The number of mutations is reported as per site per spore.

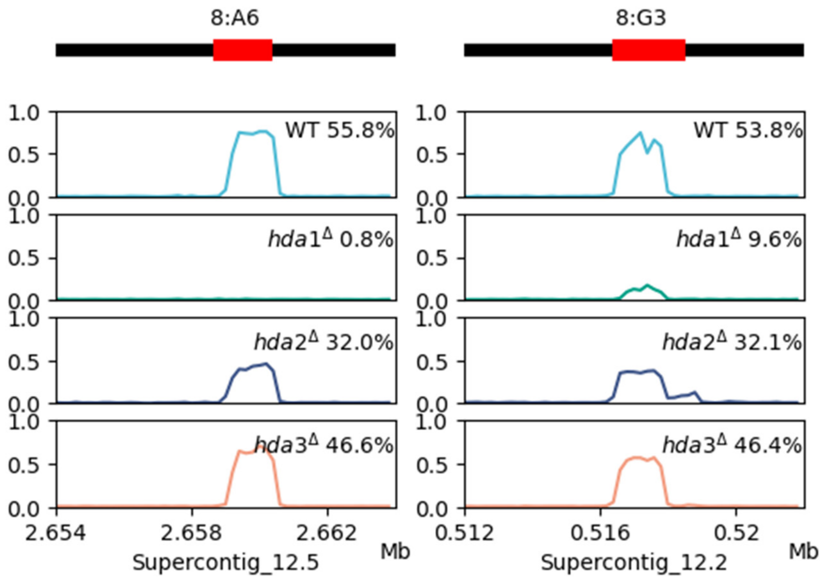

Supplementary Figure S6. Distinct influences of HDA-1 and HDA-2/3 on methylated components in prior studies. In prior studies with Southern hybridization, methylation of component 8:A6 showed a "striking loss" in the *hda-1* mutant, while component 8:G3 exhibited a "partial loss", with *hda-2* and *hda-3* mutants showing patterns similar to WT (1). In our WGBS data, the methylation levels of these components in the *hda-1* mutant decreased much greater than in the *hda-2* and *hda-3*, consistent with previous reports. However, though more subtle, methylation levels in *hda-2* and *hda*-3 also decreased compared to WT.

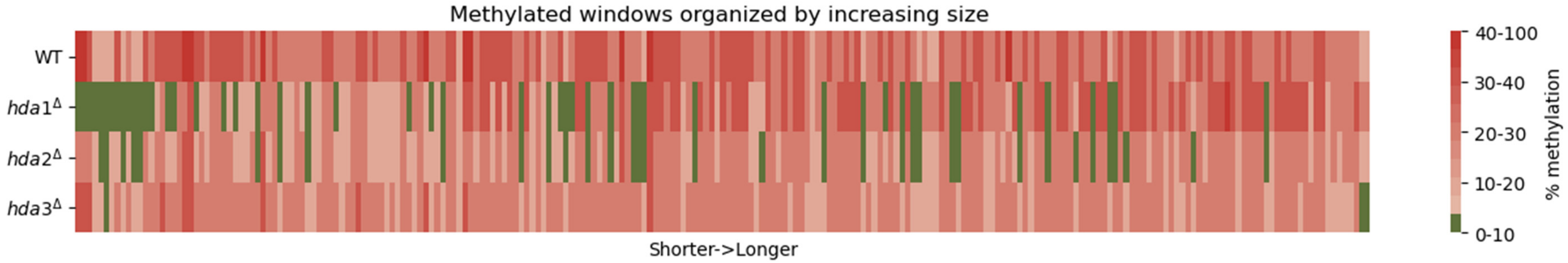

Supplementary Figure S7. Changes in 5mC methylation levels after knocking out HDACs. Heat map analysis showing the relative levels of 5mC for all methylated regions in WT, sorted from shortest to longest region. Methylated regions in WT were defined as described by Honda et al (2). To assess the changes in methylation levels in each region after knocking out HDACs, the genome was divided into 100bp windows, and the relative methylation level for each window was quantified as 5mCs/(Cs+5mCs). The relative methylation level for each mutant at the corresponding methylated region in WT was determined by averaging the values of overlapping windows in the analyzed mutant. This figure, inspired by Honda et al (2), illustrates similar patterns in *hda-1* mutants and highlights distinct effects between HDA-1 and HDA-2/3.

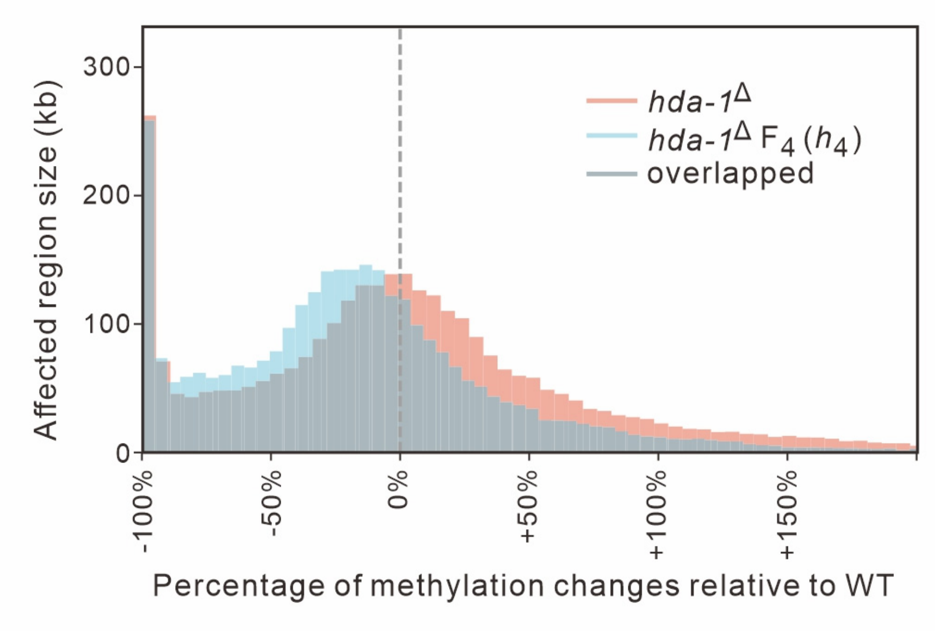

Supplementary Figure S8. Cytosine methylation changes in *hda-1* mutants at parental and F_4 generation strains. Histograms showing the distribution of percentage of methylation changes relative to WT in *hda-1* parental and F_4 generation strains (red for *hda-1*$^{\Delta}$, green for *hda-1*$^{\Delta}$ F_4 and gray for the overlapped area). Methylation change were quantified in 100bp windows. Most of the windows show a relatively mild decrease after four crosses.

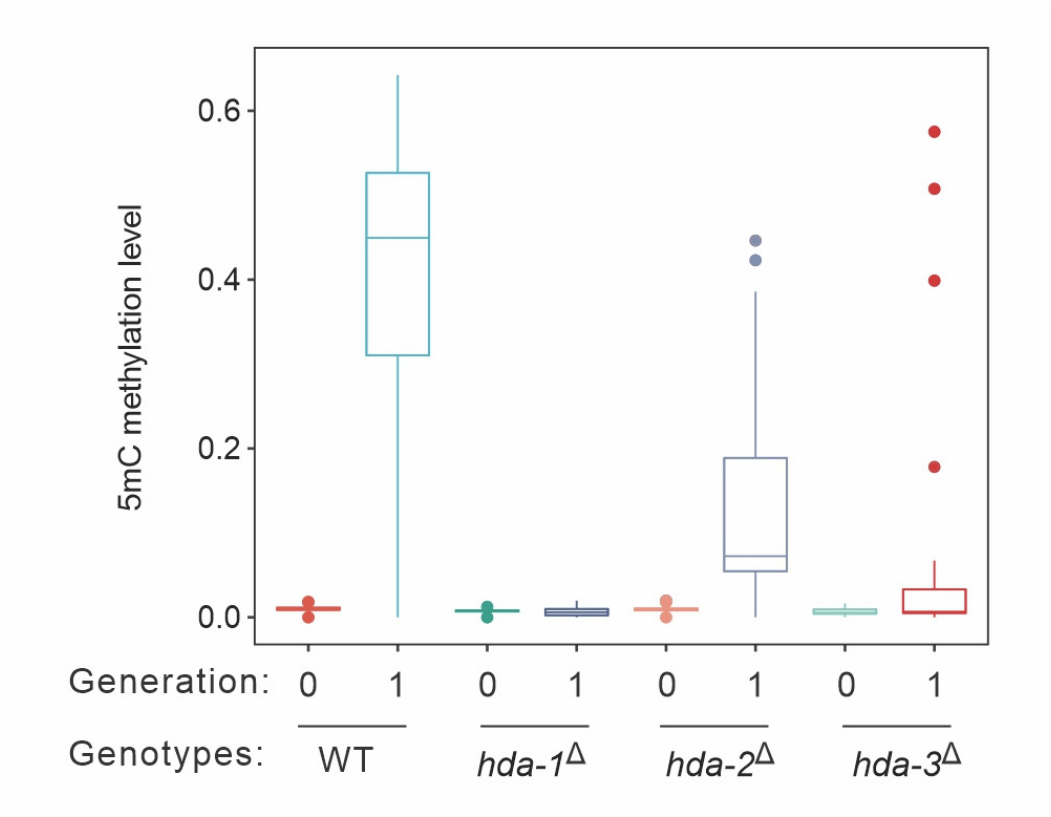

Supplementary Figure S9. DNA methylation level on *Sly1-1*in wild-type (WT), *hda-1*, *hda-2* and *hda-3* knockout strains. The 5mC methylation level on *Sly1-1* was quantified in 500 bp windows.

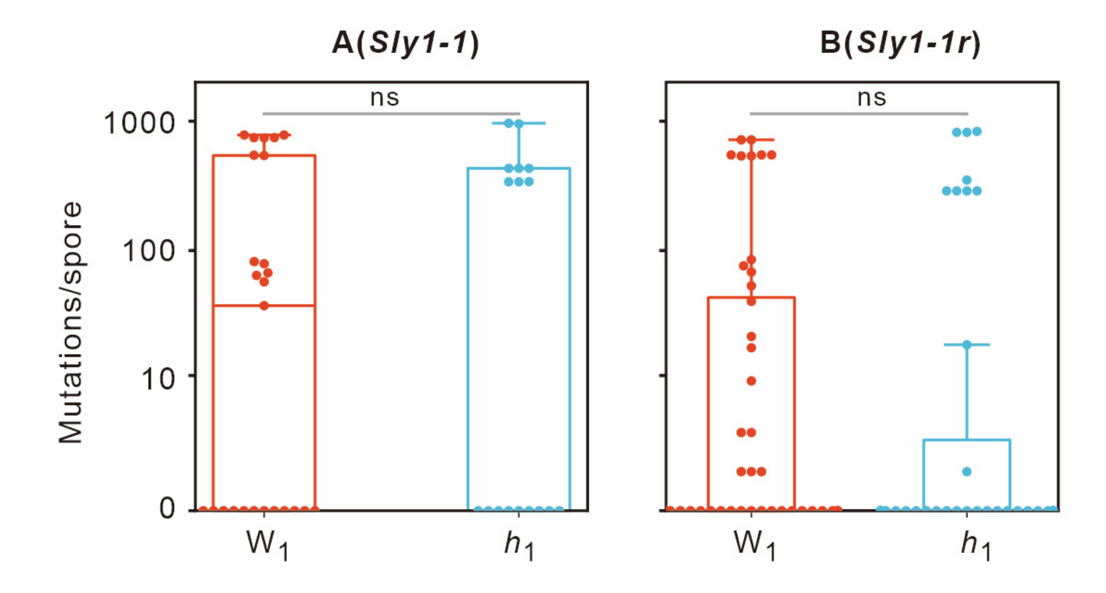

Supplementary Figure S10. Number of RIP mutations observed on *Sly1* copies in F_1 progeny with or without *hda-1.* Cross progeny of *hda-1*$^+$ strains: W_1, cross progeny of *hda-1*$^\Delta$ strains: h_1. ns, no statistical significance (Brunner-Munzel tests, $P = 0.99$ for *Sly1-1* and $P = 0.24$ for *Sly1-1r*).

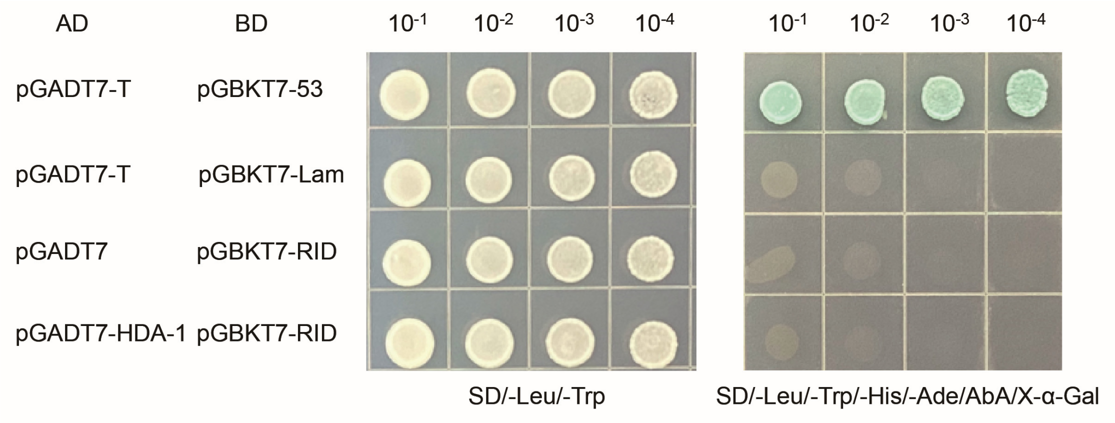

Supplementary Figure S11. No evidence of direct interaction between RID and HDA-1. The interaction between RID and HDA-1 was tested using a yeast two-hybrid system. RID was fused to the DNA binding domain (DBD) as the bait, while HDA-1 was fused to the activation domain (AD) as the prey. The plasmids were transformed into Y2HGold yeast cells, and transformants were plated at dilutions of 10^{-1}, 10^{-2}, 10^{-3} and 10^{-4} on synthetically defined (SD) medium lacking leucine (Leu) and tryptophan (Trp) (SD/-Leu/-Trp), as well as on SD medium lacking Leu, Trp, histidine (His) and adenine, but containing Aureobasidin A (AbA) and X-α-Gal (SD/-Leu/-Trp/-His/-Ade/ AbA/ X-α-Gal). Co-transformation with pGADT-7 and pGBKT7-53 served as a positive control, and the presence of blue colonies indicated a positive interaction. In contrast, co-transformation with pGADT-7 and pGBKT7-Lam was used as a negative control.

Supplementary Tables

Supplementary Table S1. *Neurospora crassa* strains used in this study

Strain	Mating type	*Sly1* type	Genotype	Source
FGSC2489	A	A	Wild type	Tian Chaoguang, Tianjin Institute of Industrial Biotechnology, Chinese Academy of Sciences
FGSC3246	a	0	Wild type	The Fungal Genetics Stock Center
ku70^{RIP}	a	B	*mus-51*^{RIP}	Qun He, School of biology, China Agricultural University
WT^{AB}	A	AB	*mus-51*^{RIP}	Cross progeny of FGSC2489 and *ku70*^{RIP}
WT^{0}	a	0	*mus-51*^{RIP}	Cross progeny of FGSC2489 and *ku70*^{RIP}
WT^{B}-A	A	B	*mus-51*^{RIP}	Cross progeny of FGSC2489 and *ku70*^{RIP}
R-L-R-WT	A	B	R-L-R, *mus-51*^{RIP}	WT^{B}-A transformed with R-L-R
R-L-R'-WT	a	B	R-L-R', *mus-51*^{RIP}	*ku70*^{RIP} transformed R-L-R'
R-L-R"-WT	a	B	R-L-R", *mus-51*^{RIP}	*ku70*^{RIP} transformed R-L-R"
W$_1$	A	AB	*mus-51*^{RIP}	Cross progeny of WT^{AB} and *ku70*^{RIP}
W$_2$	A	AB	*mus-51*^{RIP}	Cross progeny of W$_1$ and WT^{0}
W$_3$	A	AB	*mus-51*^{RIP}	Cross progeny of W$_2$ and WT^{0}
W$_4$	A	AB	*mus-51*^{RIP}	Cross progeny of W$_3$ and WT^{0}
dim-2$^{\Delta}$-A	A	B	*dim-2*$^{\Delta}$, *mus-51*^{RIP}	WT^{B}-A transformed with *dim-2*5flank::*hph*$^{+}$-*dim-2*3flank
dim-2$^{\Delta}$-a	a	B	*dim-2*$^{\Delta}$, *mus-51*^{RIP}	*ku70*^{RIP} transformed with *dim-2*5flank::*hph*$^{+}$-*dim-2*3flank
dim-2^{AB}	A	AB	*dim-2*$^{\Delta}$, *mus-51*^{RIP}	WT^{AB} transformed with *dim-2*5flank::*hph*$^{+}$-*dim-2*3flank
R-L-R-*dim-2*$^{\Delta}$	A	B	R-L-R, *dim-2*$^{\Delta}$, *mus-51*^{RIP}	*dim-2*$^{\Delta}$-A transformed with R-L-R
R-L-R'-*dim-2*$^{\Delta}$	a	B	R-L-R', *dim-2*$^{\Delta}$, *mus-51*^{RIP}	*dim-2*$^{\Delta}$-a transformed with R-L-R'
R-L-R"-*dim-2*$^{\Delta}$	a	B	R-L-R", *dim-2*$^{\Delta}$, *mus-51*^{RIP}	*dim-2*$^{\Delta}$-a transformed with R-L-R"

rid$^{\Delta}$-A	A	B	*rid*$^{\Delta}$, *mus-51*^{RIP}	WT^{B}-A transformed with *rid*5flank::*hph*$^{+}$-*rid*3flank
rid$^{\Delta}$-a	a	B	*rid*$^{\Delta}$, *mus-51*^{RIP}	*ku70*^{RIP} transformed with *rid*5flank::*hph*$^{+}$-*rid*3flank
rid^{AB}	A	AB	*rid*$^{\Delta}$, *mus-51*^{RIP}	WT^{AB} transformed with *rid*5flank::*bar*$^{+}$-*rid*3flank
rid^{0}	a	0	*rid*$^{\Delta}$, *mus-51*^{RIP}	WT^{0} transformed with *rid*5flank::*hph*$^{+}$-*rid*3flank
R-L-R-*rid*$^{\Delta}$	A	B	R-L-R, *rid*$^{\Delta}$, *mus-51*^{RIP}	*rid*$^{\Delta}$-A transformed with R-L-R
R-L-R'-*rid*$^{\Delta}$	a	B	R-L-R', *rid*$^{\Delta}$, *mus-51*^{RIP}	*rid*$^{\Delta}$-a transformed with R-L-R'
R-L-R''-*rid*$^{\Delta}$	a	B	R-L-R'', *rid*$^{\Delta}$, *mus-51*^{RIP}	*rid*$^{\Delta}$-a transformed with R-L-R''
W_1^{rid}	A	AB	*rid*$^{\Delta}$, *mus-51*^{RIP}	W_1 transformed with *rid*5flank::*bar*$^{+}$-*rid*3flank
dim-2$^{\Delta}$*rid*$^{\Delta}$-a	a	B	*dim-2*$^{\Delta}$, *rid*$^{\Delta}$, *mus-51*^{RIP}	Cross progeny of *dim-2*$^{\Delta}$ and *rid*$^{\Delta}$
dim-2$^{\Delta}$*rid*$^{\Delta}$-A	A	B	*dim-2*$^{\Delta}$, *rid*$^{\Delta}$, *mus-51*^{RIP}	Cross progeny of *dim-2*$^{\Delta}$ and *rid*$^{\Delta}$
R-L-R-*dim-2*$^{\Delta}$*rid*$^{\Delta}$	A	B	R-L-R, *dim-2*$^{\Delta}$, *rid*$^{\Delta}$, *mus-51*^{RIP}	*dim-2*$^{\Delta}$*rid*$^{\Delta}$-A transformed with R-L-R
hda-1$^{\Delta}$-a	a	B	*hda-1*$^{\Delta}$, *mus-51*^{RIP}	*ku70*^{RIP} transformed with *hda-1*5flank::*hph*$^{+}$-*hda-1*3flank
hda-1^{AB}	A	AB	*hda-1*$^{\Delta}$, *mus-51*^{RIP}	WT^{AB} transformed with *hda-1*5flank::*hph*$^{+}$-*hda-1*3flank
hda-1^{0}	a	0	*hda-1*$^{\Delta}$, *mus-51*^{RIP}	WT^{0} transformed with *hda-1*5flank::*hph*$^{+}$-*hda-1*3flank
W_1^{hda-1}	A	AB	*hda-1*$^{\Delta/\Delta}$, *mus-51*^{RIP}	W_1 transformed with *hda-1*5flank::*hph*$^{+}$-*hda-1*3flank
$W_1^{rid;hda-1}$	A	AB	*rid*$^{\Delta}$, *hda-1*$^{\Delta}$, *mus-51*^{RIP}	W_1 transformed with *hda-1*5flank::*hph*$^{+}$-*hda-1*3flank and *rid*5flank::*bar*$^{+}$-*rid*3flank
h_1	A	AB	*hda-1*$^{\Delta}$, *mus-51*^{RIP}	Cross progeny of *hda-1*^{AB} and *hda-1*$^{\Delta}$-a
h_2	A	AB	*hda-1*$^{\Delta}$, *mus-51*^{RIP}	Cross progeny of h_1 and *hda-1*^{0}
h_3	A	AB	*hda-1*$^{\Delta}$, *mus-51*^{RIP}	Cross progeny of h_2 and *hda-1*^{0}
h_4	A	AB	*hda-1*$^{\Delta}$, *mus-51*^{RIP}	Cross progeny of h_3 and *hda-1*^{0}
$h1^{HDA-1}$	A	AB	*mus-51*^{RIP}	h_1 transformed with *hda-1*::*bar*$^{+}$-*hda-1*3flank
hda-2$^{\Delta}$-a	a	B	*hda-2*$^{\Delta}$, *mus-51*^{RIP}	*ku70*^{RIP} transformed with *hda-2*5flank::*hph*$^{+}$-*hda-2*3flank
hda-2^{AB}	A	AB	*hda-2*$^{\Delta}$, *mus-51*^{RIP}	WT^{AB} transformed with *hda-2*5flank::*hph*$^{+}$-*hda-2*3flank
hda-3$^{\Delta}$-a	a	B	*hda-3*$^{\Delta}$, *mus-51*^{RIP}	*ku70*^{RIP} transformed with *hda-3*5flank::*hph*$^{+}$-*hda-3*3flank

hda-3^{AB}	A	AB	*hda-3*$^{\Delta}$, *mus-51*^{RIP}	WT^{AB} transformed with *hda-3*5flank::*hph*$^{+}$-*hda-3*3flank
LexAO only	A	B	*his-3*$^{+}$::*hph*$^{+}$-LexAO, *mus-51*^{RIP}	WT^{B}-A transformed with his-3^{+}::*hph*$^{+}$-LexAO
HP1-LexADBD-a	a	B	*his-3*$^{+}$::*hph*$^{+}$-LexAO, *hpo*–LexADBD::*bar*$^{+}$, *mus-51*^{RIP}	*ku70*^{RIP} transformed with *his-3*$^{+}$::*hph*$^{+}$-LexAO and *hpo*–LexADBD::*bar*$^{+}$
HP1-LexADBD-A	A	B	*his-3*$^{+}$::*hph*$^{+}$-LexAO, *hpo*–LexADBD::*bar*$^{+}$, *mus-51*^{RIP}	WT^{B}-A transformed with *his-3*$^{+}$::*hph*$^{+}$-LexAO and *hpo*–LexADBD::*bar*$^{+}$
RID-LexADBD-a	a	B	*his-3*$^{+}$::*hph*$^{+}$-LexAO, *rid*–LexADBD::*bar*$^{+}$, *mus-51*^{RIP}	*ku70*^{RIP} transformed with *his-3*$^{+}$::*hph*$^{+}$-LexAO and *rid*–LexADBD::*bar*$^{+}$
RID-LexADBD-A	A	B	*his-3*$^{+}$::*hph*$^{+}$-LexAO, *rid*–LexADBD::*bar*$^{+}$, *mus-51*^{RIP}	WT^{B}-A transformed with *his-3*$^{+}$::*hph*$^{+}$-LexAO and *rid*–LexADBD::*bar*$^{+}$
RID-Histag-A	A	B	*rid*–6×His::*hph*$^{+}$, *mus-51*^{RIP}	*ku70*^{RIP} transformed with *rid*–6×His::*hph*$^{+}$
RID-Histag-a	a	B	*rid*–6×His::*hph*$^{+}$, *mus-51*^{RIP}	WT^{B}-A transformed with *rid*–6×His::*hph*$^{+}$

Supplementary Table S2. Primers used in this study

Primer	Sequence (5' - 3')
**a. For *rid*-KO**	
rid-5flankF	GAGAGGGAAAATGGGAATG
rid-5flankR	GCTCTAGACTGGTCGGCAACTACAGATA
rid-3flankF	GCTCTAGAGCAGCAGATTTGGGTCATT
rid-3flankR	GGATGTCAAGGGCACTATT
*rid-hph*F	CTGTAGTTGCCGACCAGTCTAGAGCGAAAGGCGGACAGGTATC
*rid-hph*R	TGACCCAAATCTGCTGCTCTAGAGCTTCTGGGTAAACGACTCATAG
**b. For *dim-2*-KO**	
dim-2-5flankF	CACACAGAACCCTAAAGAAAC
dim-2-5flankR	CCCAAGCTTGCTTGGCTGATAACGAACT
dim-2-3flankF	CGGGATCCGTTGTAGTGCGACGAGGTA
dim-2-3flankR	TGCCCTGCTAACAAAGAC
*dim-2-hph*F	TCGTTATCAGCCAAGCAAGCTTGGGGAAAGGCGGACAGGTATC
*dim-2-hph*R	TCGTCGCACTACAACGGATCCCGTTCTGGGTAAACGACTCATAG
**c. For *hda-1*-KO**	
hda-1-5flankF	CTATCCTTCGGCATTGTTC
hda-1-5flankR	CTACTGGGCTGCTTCCTAAGCTCTAGAGCGGTGCTGCGGTAGAAATAA
hda-1-3flankF	TTCCACACAACATACGAGCCGCTCTAGAGCTTGGTGATGGCGAGTTTTA
hda-1-3flankR	GGCGGTTGTTGGTGAAGTA
*hda-1-hph*F	GCTCTAGAGCTTAGGAAGCAGCCCAGTAG
*hda-1-hph*R	GCTCTAGAGCGGCTCGTATGTTGTGTGGAA
**d. For *hda-2*-KO**	
hda-2-5flankF	GCGTTCAATCACTTCCACA

hda-2-5flankR	CTACTGGGCTGCTTCCTAAGCTCTAGAGCGTCTAGGAATGCCGTGAAA
hda-2-3flankF	TTCCACACAACATACGAGCCGCTCTAGAGCTTCCTGATGATGCCCCTAC
hda-2-3flankR	GCAAAGCCTCCAAATCAGT
*hda-2-hph*F	GCTCTAGAGCTTAGGAAGCAGCCCAGTAG
*hda-2-hph*R	GCTCTAGAGCGGCTCGTATGTTGTGTGGAA
**e. For *hda-3*-KO**	
hda-3-5flankF	GGCGGTTTGTTGTTTAGGT
hda-3-5flankR	GCTCTAGAGCCGTTGTGTTGTGTTGCGTT
hda-3-3flankF	TTCCACACAACATACGAGCCGCTCTAGAGCCCCCGAACAAACACTCAGT
hda-3-3flankR	GGCAATCCCACTCAAGGTA
*hda-3-hph*F	GCTCTAGAGCTTAGGAAGCAGCCCAGTAG
*hda-3-hph*R	GCTCTAGAGCGGCTCGTATGTTGTGTGGAA
**f. For R-L-R constructs**	
csr-5flankF	CCTCCCCAACTCCGAAAT
csr-5flankR	CCATTTAAATCACGTGAGGCCTCCTAGGCTTAAGCCGCGGGCACACCCACCCTTCTTC
csr-repeat1F	AAGCCTAGGAGGCCTCACGTGATTTAAATGGTAGTAAGGTAGGAGAAGGAGCATATGTCAGGCTTCAAAGAGCTGAG
csr-repeat1R	AATTCCTGCAGCCCGGGGGATCCACTAGTTCTAGAGCGGCCGCCCCTAACCTCTAAACCT CTTTCCACAAGGAGGGAAACGCAAGT
csr-repeat2F	AGTGGATCCCCCGGGCTGCAGGAATTCGATATCAAGCTTATCGATACCGTCGACCTCGAGGGGGGGCCCTGCAGCCAT TGACGACATT
csr-3flankR	CGAAATGCCCGTGAACTT
**g. For ChIP-qPCR**	
Positive-F	CTAGCGTCCAAAGGTCTA
Positive-R	TATTATTCCGTTCCCTATTTAGC
Negative-F	CATCGGAATCTCGTCTTG
Negative-R	ACTGTGTAGAAGCCTGAT

Sly1-1-F	AACGCCAGCAATATCAAC
Sly1-1-R	GTCCAGCAGAATAGTAGGT
**h. For RID-LexADBD**	
*rid*TAA5flankF	CCACTTCCCTATACGGTCACA
*rid*TAA5flankR	CTTCTTCTTGGGACCGCCGCCACCGCCACCGCCGCCGTCGTCGAAAAGCTCCAT
rid-LexADBDF	GGCGGCGGTGGCGGTGGCGGCGGTCCCAAGAAGAAG
rid-LexADBDR	GCTCTAGAGCTTAGGCGTTGGGCTTGAAGA
*rid-bar*F	TCTTCAAGCCCAACGCCTAAGCTCTAGAGCCACCAAACCGTCAAGATGT
*rid-bar*R	CTCTAGAGCTATTACCGCCTTTGAGTGAG
*rid*TAA3flankF	CTCACTCAAAGGCGGTAATAGCTCTAGAGTAAAGTCTTGATCCCCAAAG
*rid*TAA3flankR	TGCTATGGAGGCGGAACT
**i. For HP1-LexADBD**	
*hpo*TAG5flankF	TCACACCAGCTCATAAAAATGCCG
*hpo*TAG5flankR	TTCTTCTTGGGACCGCCGCCACCGCCACCGCCGCCTTGCGAGACGCTGCCCTCGCGATC
hpo-LexADBDF	GGCGGCGGTGGCGGTGGCGGCGGTCCCAAGAAGAAG
hpo-LexADBDR	GCTCTAGAGCCTAGGCGTTGGGCTTGAAGA
*hpo-bar*F	TCTTCAAGCCCAACGCCTAGGCTCTAGAGCCACCAAACCGTCAAGATGT
*hpo-bar*R	CTCTAGAGCTATTACCGCCTTTGAGTGAG
*hpo*TAG3flankF	CTCACTCAAAGGCGGTAATAGCTCTAGAGAGAAGACCGAGGTAGCACTTCTCGAA
*hpo*TAG3flankR	AGAGAGCCGCAAGGCTCAGGGACT
**j. For *his-3*3flank-LexAO**	
his-3-35flankF	AGGCTATTGAGGACGAGGTT
his-3-35flankR	CTACTGGGCTGCTTCCTAAGCTCTAGAGCAAGACACCATTTCCCACAC
his-3-3*hph*F	GCTCTAGAGCTTAGGAAGCAGCCCAGTAG
LexAO-*hph*R	CTGTTTTTTTATACAGCTGTTTTTTTATACAGCTGTTTTTTTATACAGCTGTTTTTTTATACAGCGGCTCGTATGTTGTGTG
his-3-33flankF	CTGTATAAAAAAACAGCTGTATAAAAAAACAGGTTGACATTGACTGGGACAC

his-3-33flankR	CGTTATTGCCGTTTGACC
**k. For RID-His**	
*rid*his-5flankF	CCACTTCCCTATACGGTCACA
*rid*his-5flankR	TTAATGGTGATGGTGATGATGGGATCCGTCGTCGAAAAGCTCCAT
*rid*his-*hph*F	GGATCCCATCATCACCATCACCATTAATTAGGAAGCAGCCCAGTAG
*rid*his-*hph*R	GCTCTAGAGCCGGCTCGTATGTTGTGTG
*rid*his-3flankF	CACACAACATACGAGCCGGCTCTAGAGCTAAAGTCTTGATCCCCAAAG
*rid*his-3flankR	TGCTATGGAGGCGGAACT

Supplementary Table S3. Mutations in the artificially constructed R-L-R region.

Genotype of both parents	No. of spores	SNPs in R-L-R per spore		C->T
		Repeats	Linker	
Wild type (*rid*$^{+}$; *dim-2*$^{+}$)	12	39.6	2.1	100%
rid$^{\Delta}$; *dim-2*$^{+}$	20	0.3	5.1	100%
rid^{+}; *dim-2*$^{\Delta}$	15	20	0.1	100%
rid$^{\Delta}$; *dim-2*$^{\Delta}$	11	0	0	0

Supplementary Table S4. Genomic mutations in wild-type and DNA methyltransferase mutants.

Genotype of both parents	No. of tetrads	No. of spores	No. of SNP mutations per spore (C->T%)	Dup		Non-dup	
				Clustered SNPs	Singleton SNPs	Clustered SNPs	Singleton SNPs
Wild type (*rid*$^{+}$; *dim-2*$^{+}$)	28	169	57.7 (83%)	29.9	15.9	4.6	7.2
rid$^{\Delta}$; *dim-2*$^{+}$	9	56	19.2 (93%)	9.3	5.2	2.4	2.3
rid^{+}; *dim-2*$^{\Delta}$	10	65	9.2 (84%)	4.2	4	0	1
rid$^{\Delta}$; *dim-2*$^{\Delta}$	7	44	0.7 (34%)	0	0.5	0	0.2

Supplementary Table S5. Mutations per spore per Mb on duplicates with different physical distances.

		Duplicate pairs* on same chromosome			Duplicate pairs* on different chromosomes
		Distance <5k	5k< distance <300k	Distance >300 k	
Spanned length（Mb）		0.37	1.98	3.12	5.59
Average number of mutations per spore per Mb	WT × WT	3.95	3.81	4.17	4.09
	rid$^{\Delta}$ × *rid*$^{\Delta}$	0.6	1.63	1.43	1.76
	dim-2$^{\Delta}$ × *dim-2*$^{\Delta}$	0.94	1.41	1.96	1.55
	rid$^{\Delta}$; *dim-2*$^{\Delta}$ × *rid*$^{\Delta}$; *dim-2*$^{\Delta}$	0	0.07	0.02	0.08

*All possible duplicate pairs were considered and mutations can be double-counted in different category.

Supplementary Table S6. Mutations per spore on duplicates and non-duplicates with different DNA methylation status.

		Duplicates		Non-duplicates	
Methylation level		>10%	<10%	>10%	<10%
Spanned length (Mb)		4.12	1.72	0.63	34
Average number of mutations per spore	WT × WT	21.61	2.52	5.59	6.16
	rid$^{\Delta}$ × *rid*$^{\Delta}$	9.94	0.89	7.28	4.33
	dim-2$^{\Delta}$ × *dim-2*$^{\Delta}$	8.25	0.45	0.1	0.9
	rid$^{\Delta}$; *dim-2*$^{\Delta}$ × *rid*$^{\Delta}$; *dim-2*$^{\Delta}$	0.29	0.14	0	0.14

Supplementary Table S7. Mutations identified in *Sly1-1* in progeny from crosses of different parental genotypes.

Parental genotypes	No. of tetrads	No. of spores	SNPs per spore	C->T%
WT × WT	9	44	211.2	100%
rid$^{\Delta}$ × *rid*$^{\Delta}$	15	15	1	100%
dim-2$^{\Delta}$ × *dim-2*$^{\Delta}$	15	15	237.2	100%

Supplementary Table S8. HDACs identified in RID-associated proteins with Ni-NTA purification and mass spectrometry.

Gene	Protein	MW[a] (kDa)	Protein Coverage[b] (%)	Peptides[c] (95%)
NCU02034	RID	93.54	92.43	11
NCU01525	HDA-1	83.71	5.78	1
NCU02795	HDA-2	56.52	5.81	2
NCU00824	HDA-3	72.26	14.1	4

[a]MW: The predicted protein molecular weight. [b]Protein Coverage: The percent coverage calculated by dividing the number of amino acids in all found peptides by the total number of amino acids in the entire protein sequence. [c]Peptides (95%): The number of peptide sequences in the protein group with a 95% confidence level.

Supplementary Table S9. Comparison of 5mC methylation changes relative to WT after knockout HDACs. Wilcoxon one-sample test was used to compare the mean values of altered 5mC methylation levels relative to WT, with statistical comparison made to zero to assess whether the changes were significantly different from no change (i.e., whether the mean deviated from zero).

bin size	mutant	Mean of methylation changes relative to WT (%)	*P*-value
25	*hda-1*	-0.46	3.32E-36
	hda-2	-24.18	0
	hda-3	-24.3	0
50	*hda-1*	3.07	6.50E-135
	hda-2	-22.28	0
	hda-3	-22.53	0
100	*hda-1*	4.66	4.04E-151
	hda-2	-21.77	0
	hda-3	-22.18	0
200	*hda-1*	5.7	1.74E-122
	hda-2	-21.96	0
	hda-3	-22.35	0

Supplementary References

1. Smith,K.M., Dobosy,J.R., Reifsnyder,J.E., Rountree,M.R., Anderson,D.C., Green,G.R. and Selker,E.U. (2010) H2B- and H3-Specific Histone Deacetylases Are Required for DNA Methylation in Neurospora crassa. *Genetics*, **186**, 1207–1216.

2. Honda,S., Bicocca,V.T., Gessaman,J.D., Rountree,M.R., Yokoyama,A., Yu,E.Y., Selker,J.M.L. and Selker,E.U. (2016) Dual chromatin recognition by the histone deacetylase complex HCHC is required for proper DNA methylation in Neurospora crassa. *Proc. Natl. Acad. Sci.*, **113**, E6135–E6144.
